# Recommendations and evidence for reporting items in pediatric clinical trial protocols and reports: two systematic reviews

**DOI:** 10.1186/s13063-015-0954-0

**Published:** 2015-09-18

**Authors:** April V. P. Clyburne-Sherin, Pravheen Thurairajah, Mufiza Z. Kapadia, Margaret Sampson, Winnie W. Y. Chan, Martin Offringa

**Affiliations:** The Hospital for Sick Children, Peter Gilgan Centre for Research and Learning, SickKids Research Institute, Child Health Evaluative Sciences, 686 Bay Street, Toronto, ON M5G 0A4 Canada; Children’s Hospital of Eastern Ontario, 401 Smyth Road, Ottawa, ON K1H 8L1 Canada; Senior Scientist and Program Head Child Health Evaluative Sciences, The Hospital for Sick Children, Peter Gilgan Centre for Research and Learning, SickKids Research Institute, 686 Bay Street, Toronto, ON M5G 0A4 Canada

**Keywords:** Clinical trials, Protocols, Systematic review, Reporting, SPIRIT, SPIRIT-C, CONSORT, CONSORT-C

## Abstract

**Background:**

Complete and transparent reporting of clinical trial protocols and reports ensures that these documents are useful to all stakeholders, that bias is minimized, and that the research is not wasted. However, current studies repeatedly conclude that pediatric trial protocols and reports are not appropriately reported. Guidelines like SPIRIT (Standard Protocol Items: Recommendations for Interventional Trials) and CONSORT (Consolidated Standards of Reporting Trials) may improve reporting, but do not offer guidance on issues unique to pediatric trials. This paper reports two systematic reviews conducted to build the evidence base for the development of pediatric reporting guideline extensions: 1) SPIRIT-Children (SPIRIT-C) for pediatric trial protocols, and 2) CONSORT-Children (CONSORT-C) for pediatric trial reports.

**Method:**

MEDLINE, the Cochrane Methodology Register, and reference lists of included studies were searched. Publications of any type were eligible if they included explicit recommendations or empirical evidence for the reporting of potential items in a pediatric protocol (SPIRIT-C systematic review) or trial report (CONSORT-C systematic review). Study characteristics, recommendations and evidence for pediatric extension items were extracted. Recurrent themes in the recommendations and evidence were identified and synthesized. All steps were conducted by two reviewers.

**Results:**

For the SPIRIT-C and CONSORT-C systematic reviews 366 and 429 publications were included, respectively. Recommendations were identified for 48 of 50 original reporting items and sub-items from SPIRIT, 15 of 20 potential SPIRIT-C reporting items, all 37 original CONSORT items and sub-items, and 16 of 22 potential CONSORT-C reporting items. The following overarching themes of evidence to support or refute the utility of reporting items were identified: transparency; reproducibility; interpretability; usefulness; internal validity; external validity; reporting bias; publication bias; accountability; scientific soundness; and research ethics.

**Conclusion:**

These systematic reviews are the first to systematically gather evidence and recommendations for the reporting of specific items in pediatric protocols and trials. They provide useful and translatable evidence on which to build pediatric extensions to the SPIRIT and CONSORT reporting guidelines. The resulting SPIRIT-C and CONSORT-C will provide guidance to the authors of pediatric protocols and reports, respectively, helping to alleviate concerns of inappropriate and inconsistent reporting, and reduce research waste.

**Electronic supplementary material:**

The online version of this article (doi:10.1186/s13063-015-0954-0) contains supplementary material, which is available to authorized users.

## Background

High-quality clinical trials provide the best evidence for efficacy and safety of interventions [1], and are required to improve upon our current paucity of evidence in children [1, 2]. However, researchers have an ethical obligation to only include children in trials that are appropriately conducted and reported [1–4]. Complete and transparent reporting of trial protocols and reports ensures that these documents are useful to all stakeholders [3, 4], that bias is minimized [4–7], and that the research is not wasted [3, 4]. It also allows the readers of trial protocols and reports to critically assess trial methodology, ethical considerations, and internal and external validity [8].

Systematic reviews of pediatric literature have repeatedly concluded that pediatric trial reports are not appropriately reported [3, 4, 6, 9–17]. Despite reporting guidelines for trial reports, reviewers identified gaps in reporting across pediatric specialties, including inadequate reporting of important items such as adverse events [4, 12, 13, 18], interventions [12–14, 19], outcomes [12, 20], sequence generation [12, 20], allocation concealment [12, 20], blinding [11, 12], sample size calculations [11, 12], consent and assent information [21], and many more [12, 20, 22].

Similarly, Tetzlaff et al. [23] and others [24–26] have described inappropriate reporting in trial protocols. Despite protocol-reporting guidelines, reviewers identified gaps in reporting of outcomes [24], allocation concealment [25], sample size calculations [26], and sponsor and investigator roles [27].

The SPIRIT (Standard Protocol Items: Recommendations for Interventional Trials) [28] and CONSORT (Consolidated Standards of Reporting Trials) [8] initiatives aim to harmonize and standardize reporting guidelines to tackle the problem of inappropriate reporting in trial protocols and reports, respectively. Reporting guidelines provide checklists of specific concepts, called reporting items, which need to be addressed in documents such as protocols and trial reports. SPIRIT is a protocol-reporting guideline that identifies 33 key reporting items and 17 sub-items to be addressed in all clinical trial protocols. CONSORT is a reporting guideline that identifies 25 key reporting items and 12 sub-items to be addressed in all clinical trial reports upon completion of the clinical trial. The guidelines, when followed, allow the readers of trial protocols and reports to critically assess trial methodology, ethical considerations, as well as internal and external validity. Although these guidelines may improve reporting [4], neither SPIRIT nor CONSORT offer guidance on issues unique to pediatric trials [1–3, 6, 13, 14, 29].

Scientific, ethical and safety considerations for trials with children often differ from trials with adults [2]. As it is unethical to expose children to the risks of research unless it is scientifically valid, and inadequate reporting of pediatric-specific considerations may lead to biased estimates of treatment effects [6], these considerations must be accounted for in the trial protocol and report [1, 5, 14, 21, 22, 29]. For example, growth and maturation must be considered in all aspects of pediatric trial design and interpretation [1, 3, 29] as it alters pharmacokinetics, metabolism, excretion, drug efficacy, receptors, and end-organ drug responses [2, 30–37]. Maturation and development does not happen in a linear fashion [31, 34, 38–40]. Consequently, pediatric clinical trial design requires an in-depth understanding of developmental trajectory in the eligible population during the trial period [31, 34, 38–40]. Failure to account for growth and maturation when designing a pediatric clinical trial can lead to inappropriate interventions which could result in therapeutic failure or serious adverse events in children [2, 30, 31, 33, 41, 42].

These pediatric-specific scientific, ethical and safety consideration differences have led to repeated calls for pediatric-specific reporting guidance [1–3, 6, 13, 14]. Empirically developed pediatric extensions of the SPIRIT and CONSORT guidelines are needed to ensure these issues are appropriately reported in pediatric trial protocols and reports. Failure to account for these differences in trial protocols can lead to biased or inappropriate trials [4–7], or protocols that are too ambiguous to be useful to all stakeholders [3, 4]. Failure to account for these differences in trial reports can lead to biased reporting [4–7], research that is not useful to all stakeholders [3, 4], and is, therefore, considered waste of research [3].

Pediatric extensions of the SPIRIT and CONSORT guidelines will help ensure that readers can critically assess pediatric-specific issues relating to trial methodology, ethical considerations, trial conduct, and internal and external validity of trial results [8]. In this paper we report two systematic reviews conducted to build the evidence base for the prospective development of pediatric reporting guideline extensions: SPIRIT-Children (SPIRIT-C) for pediatric trial protocols and CONSORT-Children (CONSORT-C) for pediatric trial reports. The systematic reviews to build evidence for the planned SPIRIT-C and CONSORT-C extensions will be referred to as the “SPIRIT-C systematic review” and the “CONSORT-C systematic review,” respectively. Potential reporting items to be included in each extension were identified through environmental scans of existing item recommendations [1] and Delphi studies.

### Objectives

The objectives of this SPIRIT-C systematic review are to: (1) identify empirical evidence to support or refute the importance of reporting original SPIRIT and candidate SPIRIT-C extension items, in terms of pediatric trial design, conduct, or interpretation, (2) identify recommendations for the reporting of specific SPIRIT and SPIRIT-C protocol items, and (3) identify any additional methodological and ethical issues that are specific to pediatric trials.

The objectives of this CONSORT-C systematic review are to: (1) identify empirical evidence to support or refute the importance of reporting original CONSORT and candidate CONSORT-C trial extension items, in terms of pediatric trial design, conduct, or interpretation, (2) identify recommendations for the reporting of specific CONSORT and CONSORT-C trial report items, and (3) identify any additional methodological and ethical issues that are specific to pediatric trials.

### Identification of candidate extension items

Prior to this systematic review, candidate extension items were identified in accordance with best practices for reporting guideline extension development [43]: (1) a literature review identified the initial list of potential pediatric-specific SPIRIT-C and CONSORT-C extension reporting items; (2) a Delphi exercise contributed input from diverse stakeholders on the inclusion, exclusion, and addition of new potential SPIRIT-C and CONSORT-C extension reporting items.

The results of these systematic reviews will be brought to a consensus meeting of international pediatric trial experts and stakeholders to draft pediatric extensions of the SPIRIT and CONSORT guidelines, and will provide the evidence base of studies for the development of the SPIRIT-C and CONSORT-C Statement and Explanation and Elaboration documents.

## Methods

We used the PRISMA (Preferred Reporting Items for Systematic Reviews and Meta-Analyses) reporting guideline [44] to prepare this report on our two reviews. All criteria and search terms were pre-specified, but a formal protocol was not created. An ethics approval was not required for this work.

### Eligibility criteria and definitions

Publications of any type were eligible if they included explicit recommendations for reporting in a pediatric trial protocol (for the SPIRIT-C systematic review) or trial report (for the CONSORT-C systematic review), and/or if they included empirical evidence to support or refute reporting concepts in a pediatric trial protocol or trial report.

An explicit recommendation or endorsement for the reporting of an item was regarded as a *recommendation*. An example of a statement regarded as a recommendation for the reporting of an item follows, from Smith et al.: “We argue that parental consenting procedures should be routinely reported as a methodological feature of adolescent treatment outcome studies …” [45].

Any information that supported or refuted the importance of a reporting item in pediatric clinical trials was defined as *evidence*. Our definition of evidence is consistent with the types of evidence recommended to support a reporting guideline, which includes both empirical evidence of effects or biases associated with inadequate reporting of an item and any other evidence cited to support the reporting of an item [43]. An example of a statement regarded as evidence follows, also from Smith et al.: “(Reporting of consent procedures is recommended) … given concerns about sample bias in adolescent risk behavior research when parental consent is required, (and) … to prospectively study the impact of consenting procedures on both the study participation rates and substance use reporting” [45].

A publication was considered pediatric if it included pediatric participants as defined by the authors of the publication. All publication years were eligible. Publication language was limited to English. Studies and conference proceedings published only as abstracts were excluded, as were duplicate or secondary publications.

### Information sources

Eligible publications were identified through: a systematic search of electronic databases, reference lists of articles, a systematic review of reporting guidelines by Tetzlaff et al. [23] for the SPIRIT-C systematic review, and the EQUATOR network (*E*nhancing the *QU*ality and *T*ransparency *O*f health *R*esearch) database [46]. The systematic searches were conducted in MEDLINE including in-process and non-indexed citations (1946 – Present) and adapted for The Cochrane Methodology Register (CMR) (1995 – Present), using the Ovid interface. The last searches were run on 16 May 2014 for CONSORT-C MEDLINE, 7 July 2014 for SPIRIT-C MEDLINE and CMR, and 25 July 2014 for CONSORT-C CMR. The MEDLINE searches are presented in Additional files 1 and 2. The SPIRIT-C MEDLINE and CONSORT-C MEDLINE search were amended for the SPIRIT-C CMR and CONSORT-C CMR searches, respectively. Duplicates were removed in EndNote X6 (Thomson Reuters Corporation, New York, NY, USA).

### Study selection: title and abstract screening

For each systematic review, two unblinded reviewers (ACS and PT) independently screened titles and abstracts for eligibility using the pre-defined eligibility criteria. When titles and abstracts contained insufficient information to assess whether the paper could contain recommendations or evidence as defined by the eligibility criteria, the paper was included for full-text assessment to ensure that recommendations or evidence were not missed. When the eligibility of an abstract was unclear, they were included to be re-evaluated for eligibility when reviewing full-texts during the data collection process. The result of this screening approach was that all studies addressing the pediatric population in their title or abstract were assessed in full-text as they may have contained recommendations or evidence. Abstracts that did not address the pediatric population were excluded. Disagreements on the interpretation of the eligibility criteria were resolved by consensus or by the involvement of a third reviewer (WC).

### Data collection process: full-text review

Data extraction forms were developed for each systematic review, presented in Additional file 3 for SPIRIT-C and Additional file 4 for CONSORT-C, and pilot-tested on at least 10 randomly-selected included studies. Two unblinded reviewers (ACS and PT) independently extracted data from all included studies using the data extraction forms. Disagreements on the extracted data were resolved by consensus or by the involvement of a third reviewer (WC).

### Data items

The following data were extracted from each included study, if found: study characteristics (first author, year, title, journal, study type, country, target population, trial design, and area of study based on the main topic of the publication itself); recommendation for reporting of an item; description of the recommendation (the intended study design, whether the recommendations were supported with evidence, and the level of that evidence); evidence for the reporting of an item; description of the evidence for the reporting of each item; recommendations for the reporting of items other than the existing SPIRIT or CONSORT items and SPIRIT-C or CONSORT-C candidate items; descriptions of trial methodology issues that are specific to trials with children or may deviate from adult trials; how these trial methodology issues may have been overcome within the studies; description of ethical issues that are specific to trials with children; and how these ethical issues may have been overcome. Due to the nature of the review, risks of bias of individual studies were not formally assessed.

### Thematic synthesis of results

For each systematic review, two unblinded reviewers (ACS and PT) independently analyzed the combined extracted data from all included studies using thematic synthesis method for qualitative research [47]. The synthesis was completed in three stages: i) coding of the evidence; ii) organization of the codes into descriptive themes; and iii) development of analytical themes [47]. Thematic synthesis was outside the scope of the current study and not performed on the following data items: descriptions of trial methodology issues that are specific to trials with children or may deviate from adult trials, and how these trial methodology issues may have been overcome within the studies; description of ethical issues that are specific to trials with children, and how these ethical issues may have been overcome.

#### Coding of the evidence

Evidence and recommendations for the reporting of each item were identified. The evidence from the literature for the reporting of each item was coded by two reviewers inductively by the meaning and content of the evidence [47]. For example, the following evidence for the reporting of CONSORT-C Item 2a.1: “Describe the reason to perform the clinical trial in children,” was extracted from Rothenberger et al*.*: “A solid scientific justification is an ethical requirement for studies including minors. If this justification is not satisfying, studies may be performed in adults but not in children. Ninety-one of 175 publications (52 %) gave a scientific justification for including minors, 84 publications (48 %) did not” [48]. This example of evidence was coded as “Scientific justification.” After coding the evidence, reviewers compared their codes, resolved any differences, and collaboratively created one final version of coding.

#### Descriptive and analytic themes

Similar codes were then grouped together by two independent reviewers to create descriptive themes in the meaning and content of the evidence [47]. The resulting descriptive themes were compared and differences were resolved to collaboratively create one final group of descriptive themes. One reviewer (ACS) used the descriptive themes of the evidence to infer answers to the research question: “Why recommend this pediatric-specific reporting item?” For example, evidence that was coded as “Scientific justification” was interpreted by the reviewer as answering the research question as following: “The reporting of this item ensures the inclusion of children is justified.” By interpreting the descriptive themes as answers to the research question, we were able to infer analytical themes. Similar inferred answers to our research question based on the descriptive themes of the evidence were grouped together to form analytical themes. For example, the inferred answers: “The reporting of this item ensures the inclusion of children is justified” and “The reporting of this item allows readers to assess whether informed consent and assent procedures were appropriate,” were grouped to form the analytical theme “Research ethics.” The resulting analytic themes were reviewed by another reviewer (PT) and disagreements were collaboratively resolved to create the final group of analytic themes. Studies that contained a piece of evidence to form each analytical theme were summarized to highlight the types of analytic themes supporting potential reporting items. Due to the qualitative nature of the review, publication bias and heterogeneity were not formally assessed.

## Results

**Fig. 1:**
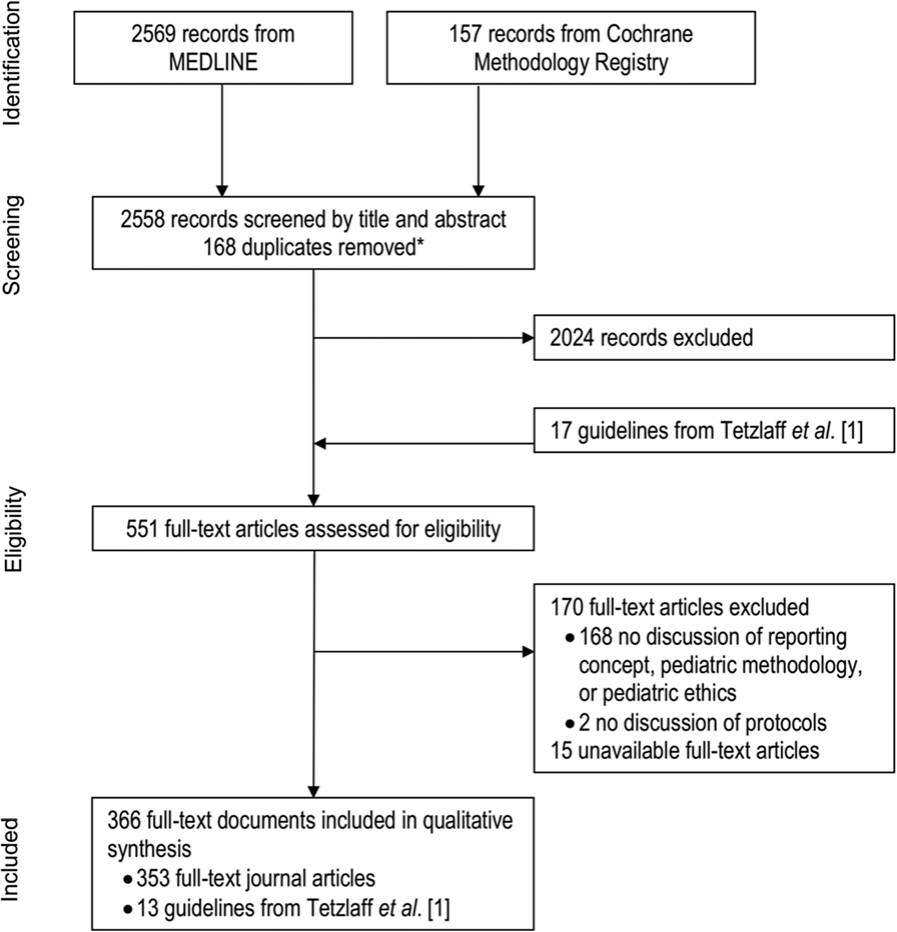
Flow of publications through the systematic review process for SPIRIT-C

A total of 2558 publications were identified through the SPIRIT-C MEDLINE and CMR database searches (Fig. 1). After screening titles and abstracts, 534 eligible full-text publications were identified. Findings from 353 relevant publications, along with 13 reporting guidelines from Tetzlaff et al. [1], were included in this review.

**Fig. 2:**
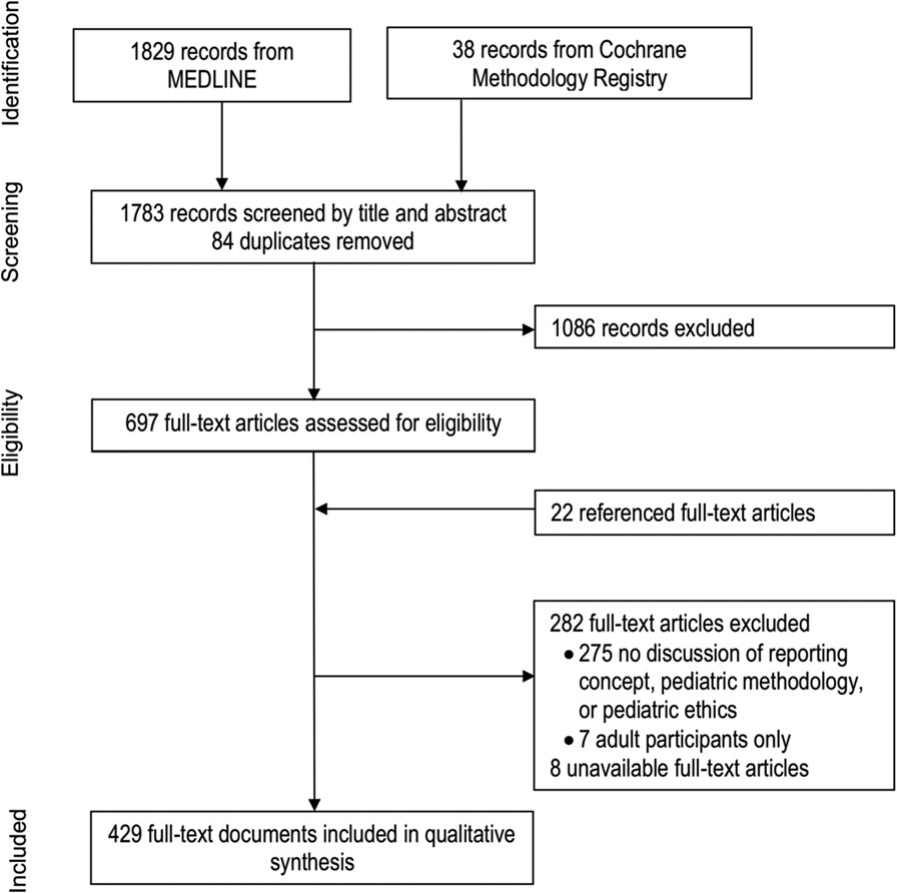
Flow of publications through the systematic review process for CONSORT-C

CONSORT-C MEDLINE and CMR database searches identified a total of 1783 publications (Fig. 2). After screening titles and abstracts, 697 eligible full-text publications were identified. Findings from 407 relevant publications, along with 22 referenced publications, were included in this review. General characteristics of the included publications for SPIRIT-C and CONSORT-C systematic reviews are shown in Tables 1 and 2.

**Table 1:** General characteristics of the included publications for the SPIRIT-C (*n* = 366) and CONSORT-C (*n* = 429) systematic reviews

	SPIRIT-C *n* (%)	CONSORT-C *n* (%)
Type of publication		
Clinical trial	21 (6)	40 (10)
Systematic review	34 (9)	92 (21)
Meta-analysis^a^	5 (1)	22 (5)
Commentary/review^b^	197 (54)	173 (40)
Cohort/cross-sectional	70 (19)	52 (12)
Protocol	23 (6)	15 (4)
Other	16 (5)	35 (8)
Target population		
Adult only	16 (4)	22 (5)
Pediatric only	271 (74)	299 (70)
All	79 (22)	108 (25)
Date of publication		
1971–1980	5 (1)	4 (1)
1981–1990	18 (5)	15 (4)
1991–2000	55 (15)	58 (13)
2001–2010	166 (45)	224 (52)
2011–2014	122 (34)	128 (30)
Presence of any *explicit recommendation* ^c^ to report an item		
Yes	92 (25)	133 (31)
No	274 (75)	296 (69)
For these explicit recommendations (*n* = 92 for SPIRIT, and *n* = 133 for CONSORT), *evidence* ^c^ identified		
Yes	54 (59)	95 (71)
No	38 (41)	38 (29)
Source of evidence for recommendations identified		
Literature review	12 (22)	29 (31)
Systematic review	5 (9)	28 (29)
Clinical trial	2 (4)	3 (3)
Consensus	9 (17)	6 (6)
Expert opinion	26 (48)	27 (29)
N/A	0 (0)	2 (2)

^a^Includes meta-analysis and systematic review

^b^Includes commentaries, editorials, opinions, and non-systematic literature reviews

^c^As defined in Method

**Table 2:** Subject foci of included publications for the SPIRIT-C (*n* = 366) and CONSORT-C (*n* = 429) systematic reviews, as identified by reviewer consensus

SPIRIT-C *n* (%)		CONSORT-C *n* (%)	
Disability	8 (2)	Alternative medicine	7 (2)
Ethics	72 (20)	Ethics	38 (9)
Evidence-based medicine	17 (5)	Evidence-based medicine	19 (3)
Infectious diseases	6 (2)	Immunology	16 (4)
Methodology	176 (48)	Infectious diseases	11 (3)
Nephrology	3 (1)	Mental health	48 (11)
Neurology	9 (2)	Methodology	150 (35)
Nutrition	5 (1)	Neurology	16 (4)
Obstetrics	2 (1)	Nutrition	6 (1)
Oncology	8 (2)	Obstetrics	12 (3)
Pharmacology	20 (5)	Oncology	18 (4)
Psychiatry	20 (5)	Pharmacology	25 (6)
Public health	6 (2)	Public health	13 (3)
Respiratory medicine	8 (2)	Respiratory medicine	21 (5)
Surgery	6 (2)	Surgery	22 (5)
		Urology	7 (2)

Publication foci are not mutually exclusive

The largest category of the publications included in these reviews was commentary or review papers, totaling 54 % of included SPIRIT-C publications and 40 % of included CONSORT-C publications. The majority of included publications addressed only the pediatric population, totaling 74 % of included SPIRIT-C publications and 70 % of CONSORT-C included publications. Only a minority of the publications contained explicit recommendations for reporting, totaling 25 % of SPIRIT-C included publications and 31 % of CONSORT-C included publications. Of these publications that contained explicit recommendations for reporting, 54 % of SPIRIT-C publications and 71 % of CONSORT-C publications provided evidence for their recommendations.

### Recommendations and evidence

Additional files 5 and 6 summarize quantitative and qualitative information regarding the presence of recommendations and/or evidence for the reporting of items in the proposed pediatric protocol and trial report, respectively. Raw and synthesized datasets for both systematic reviews are available for download and review at (http://hdl.handle.net/10864/10880) [49]. Of the 50 original reporting items and sub-items in SPIRIT, 48 were recommended for reporting by publications reviewed in systematic review; of the 20 potential extension items in SPIRIT-C, 15 were recommended. All 37 original reporting items and sub-items in CONSORT were recommended for reporting by publications reviewed, and 16 of the 22 potential extension items for CONSORT-C were recommended. No recommendation or evidence was identified that refuted the reporting of any items.

There was an average of 4.6 unique publications (standard deviation: 4.3; range: 0–17) providing recommendations for the reporting of an item in SPIRIT. An average of 16.2 unique publications (standard deviation: 17.2; range: 0–63) contributed evidence for reporting a SPIRIT item. An average of 2.6 unique publications (standard deviation: 1.9; range: 0–8) provided recommendations for an item in CONSORT. On average, 27.6 publications contributed evidence for an item (standard deviation: 13.3; range: 0–82) for CONSORT. The original SPIRIT Item 26a on consent or assent was most prominently supported with 63 publications contributing evidence and the SPIRIT-C extension Item 26a.1 on the justification for the use of proxy consent and indication of who will be eligible to provide it was well-supported with 63 publications contributing evidence (Additional file 5). Within the CONSORT-C systematic review, the original CONSORT Item 2a on scientific background and explanation of rationale was most prominently supported with 44 publications contributing evidence and the CONSORT-C extension Item 6a.1 on the validity of outcomes for the age groups was well-supported by the evidence with 82 publications contributing evidence (Additional file 6).

### Thematic synthesis

The empirical evidence identified for the reporting of specific items for pediatric trial protocols and trial reports was categorized into descriptive themes (see Additional file 7) and then into the following analytic themes: scientific soundness; research ethics; internal validity; accountability; external validity; transparency; reproducibility; interpretability; usefulness; reporting bias; and publication bias (see Table 3). Table 3 provides illustrative quotations from included studies supporting each analytic theme. For both SPIRIT-C and CONSORT-C, descriptive and analytical themes mirrored each other. The first five themes reflect scientific conduct of a clinical trial while the remainder deal with reporting of a clinical trial.

**Table 3:** Examples of study text that were categorized in each analytic theme

Analytic theme	Example of study text that was categorized within the analytic theme
Transparency	“It is our hope that more transparent reporting on consenting procedures, sample composition, and demographic characteristics would enable future studies on the association between consenting procedures and these variables, which may ultimately increase our knowledge about the impact of the unspoken rule requiring the collection of parental consent.” [45]
Reproducibility	“Other investigators should be able to replicate a vaccine trial in different settings and populations. Outcomes across trials can then be compared using: for example, the same serological cutoff points at the same intervals.” [14]
Interpretability	“Experienced practitioners and researchers also need to appreciate how setting and child characteristics affect the validity and interpretation of measures.” [158]
Usefulness	“When designing an RCT, investigators must also specify the size of the treatment effect that can be detected reliably with the proposed sample size. This minimum detectable treatment difference and the actual choice of the primary outcome strongly affect the clinical utility of the study results.” [159]
Internal validity	“Hence, it is the authors’ responsibility to demonstrate the measurement properties (validity, reliability, responsiveness) of the outcome measures used in their studies.” [160]
External validity	“As stated previously, investigators should justify such exclusions. Overly restrictive criteria concerning exclusion of patients with concurrent diagnoses seriously limit the generalizability of study results.” [161]
Reporting bias	“Selective reporting of clinical trials can also hamper the ability of conducting systematic reviews of the pediatric literature.” [89]
Publication bias	“Registration also provides a mechanism for addressing publication bias.” [162]
Accountability	“Why register trials? To satisfy public demand for unbiased evidence on the effectiveness of treatments, and to promote the public accountability of medical research in general.” [163]
Scientific soundness	“Environmental research with children should be scientifically justified, with sound research questions and valid study protocols of sufficient statistical power …” [164]
Research ethics	“In our opinion, it is very important for ethical reasons to state at least if informed consent was obtained and if the study was approved by the responsible REC.” [165]

*RCT* randomized controlled trial

The systematic reviews outlined in this paper identify recommendations and evidence that could support or refute potential reporting items for pediatric protocols and trial reports. A majority of the potential reporting items had recommendations and/or evidence identified in the literature through these reviews. Both reviews further identified methodological and ethical considerations to be taken into account when conducting and reporting pediatric trials that distinctly differ from trials in adults, which we will discuss in detail below.

### The *recommendations* for the reporting of specific items in pediatric protocols and trial reports

Our systematic reviews identified that a majority of the potential reporting items had recommendations in the literature for their reporting. These recommendations illustrate that many potential reporting items align with what these authors are already looking for in protocols and trial reports. They also suggest the need for the extension of current reporting guidelines for pediatric trials to include the pediatric-specific reporting items recommended in the articles we found in our systematic reviews. Moreover, they indicate a receptive response among the pediatric research community to the development of reporting guidelines that adopt these items.

### The empirical *evidence* for the reporting of specific items in pediatric protocols and trial reports

Similar to the recommendations, the majority of potential reporting items had supporting evidence in the included studies. The over-arching theme was the consideration of pediatric age during the design, conduct and reporting of pediatric relevant trials. The age(s) of the participating children affects all aspects of a trial, including choice of intervention and comparator, selection and measurement of relevant and valid outcomes, expected treatment effect, differences in risk/benefit profile, effects on growth and development and disease processes [21, 34, 48, 50–65]. Therefore, the detailed reporting of the age distribution of children in a trial is essential for the assessment of the appropriateness of age groups selected, the interventions, and outcomes, and the potential effects on growth and development. Within this context, three analytic themes identified in support of reporting items are summarized below, presenting some of the more prominent examples:

1. *Scientific soundness*. One analytic theme for reporting an item was to allow the reader to assess whether the pediatric protocol or trial report was scientifically sound. For example, the reporting of the effectiveness of an intervention in children, or lack thereof, allows the reader to critically evaluate whether the trial was scientifically justified. To justify a trial, authors must provide the reason to believe this intervention could be effective in children [66, 67], but that current information on this possible effectiveness is not conclusive [67–77]. Addressing known differences in pharmacokinetics and pharmacodynamics between children and adults allows the reader to evaluate whether the trial was justified and appropriate. The actual efficacy of interventions in the pediatric population may be impacted by changes in pharmacokinetics and pharmacodynamics due to maturation and development [2, 31, 32, 34, 59, 61, 66, 72, 78–87], insufficient data of high-quality and/or low-quantity [72, 73, 75, 76, 79], and incorrect predictions through the extrapolation of adult data [30, 32, 64, 65, 88–93]. Specifically, the reporting of the rationale and evidence for the comparator treatments used in the trial allow the reader to make appropriate scientific interpretations of the results [2, 30, 86, 87, 94–101]. Finally, our research found abundant support for the reporting of the validity of the outcomes used in every included age group to allow the reader to assess any potential for invalid outcome selection and measurement. Outcomes in all trials are recommended to be validated in the target population. Not all valid outcomes in adults are either relevant or valid in pediatric populations [31, 102–106] and not all child health outcomes are valid across various developmental stages within pediatrics [107–111]. Valid patient reported outcomes for adults may not be valid for young children [102–104, 112], proxy reports may differ from child experience [102, 112–116], symptoms in children may differ from those in adults, and what is considered healthy may differ between adults and children [21, 61, 106, 107, 117–122]. Also, due to these changes in outcomes measurement, validity of outcomes, and adverse impacts of treatment on the developmental trajectory of pediatric participants over time, the reporting of long-term monitoring of outcomes was also recommended [29, 34, 50, 123–131].
2. *Research ethics*. Another analytic theme for the reporting of an item was to allow the reader to assess the ethics of the trial, including whether children were protected from unnecessary research. Pediatric research should be limited to studies addressing only essential scientific questions that produce useful information [78, 118, 132]. It is not ethical to conduct research in children if comparable results can be obtained by using other methods, such as valid extrapolations from adult data or from existing data on off-label use [66, 96, 99].
  Ensuring that research does not expose participants to greater than minimal risk is an ethical requirement of pediatric research and trials must take measures to reduce risks for participants. This involves reducing the pain, discomfort, distress, and invasiveness of the procedures used [31, 32, 72, 86, 88, 132–136]. Reporting blood-sampling procedures allows the reader to critically appraise whether the volume of blood drawn in pediatric studies was minimized and sampling was infrequent as possible [32, 60, 78, 86, 87, 133, 134, 137]. Additionally, inclusion of whether a Data and Safety Monitoring Board was established enables the audience to evaluate the presence of quality and safety oversight before and during the trial [22, 86, 87, 118, 138–143]. Further, detailed reporting of the trial’s comparator treatments allows the ethical implications and risks associated with certain comparators to be assessed by the reader, like the risk of exposure to novel or off-label agents [134], the use of placebo or withholding of some standard care [61, 87, 101, 144, 145], and the inability of pediatric subjects to provide informed consent to use of some comparator groups [134, 146] make reporting of the rationale and evidence for the comparators used in the trial a necessity.
  Children may experience different harms from treatment than adults [33, 120, 147], which may not be realized until years after the trial [32, 61, 66, 72, 74, 78, 88, 96, 118, 125, 126, 132, 134, 135, 148–153]. The onset of potential harms after the conclusion of the trial, possible changes in the trial’s results due to the biological development of the participant, potential emergence of rare events later in the life of a child, and the increased ability of a child to better describe the harm due to their cognitive maturation requires that study researchers report long-term outcome monitoring procedures and how long-term safety of participants will be addressed [29, 34, 50, 123–131].
  The assent of a child, when possible, is another ethical requirement for participation in pediatric trials. Therefore, the reporting of what research information was provided to children and how assent was established was recommended as age, competence, and maturity dictates the appropriateness of the aforementioned elements [32, 61, 72, 74, 78, 88, 96, 118, 125, 126, 132, 134, 135, 148–153]. Reporting this information allows the reader to assess the ethics of the trial, by evaluating whether the information and assent process was appropriate, and whether the autonomy of the child was respected.
3. *Internal validity.* Finally, the analytic theme of internal validity was identified through our systematic reviews. Transparent reporting of certain study design, conduct, and analysis items allows the reader to assess the internal validity of the trial, including the extent to which all forms of bias were contained. As in trials outside child health, proper randomization, allocation concealment, blinding of intervention and outcome measurement, and full non- selective outcome reporting are crucial. Specifically, confounders (such as body mass index) [56, 108], assessment procedures [107–111], and test cut-off ranges may change across age and development groups. Also, the reporting of stratified randomization considerations allows the reader to assess whether the stratification variables were appropriate. The reporting of effect modification considerations allows the reader to assess the appropriateness of the considerations [87, 98], whether they were considered a priori [89, 154] and the risk of bias in the trial by evaluating whether pediatric-specific age and development-related confounding variables were accounted for [34, 70, 89, 117, 155, 156]. The reporting of the subgroup analyses allows the reader to critically assess the validity of the trial analyses by evaluating whether analyses were decided upon before or after the trial, and whether analyses were appropriate [142, 157].

## Discussion

Pediatric-specific trial protocols and trial reports need pediatric-specific guidance informed by evidence to contain bias, maximize legitimacy, and enable correct interpretation, knowledge translation, and application. Previous systematic reviews have shown inappropriate reporting of pediatric protocols [24–26] and pediatric trial reports [3, 4, 6, 9–17]. Despite repeated calls for pediatric-specific reporting guidance in the literature, there has been no previous work completed to build the evidence base to create this guidance. The recommendations and evidence we identified and summarized in this paper provide a basis for decisions to be made in a consensus meeting for the development of pediatric extensions to the current reporting guidelines. Building on the success of the SPIRIT and CONSORT guidelines, these evidence-based guidelines will help to ensure that authors who are looking to appropriately report pediatric trial protocols and trial reports have evidence-based guidance.

### Limitations and strengths

While the MEDLINE and CMR databases were selected for their relevance and comprehensiveness, our electronic search was restricted to these two databases, and to English publications only. Publications were eligible based on content rather than type, and it is possible that eligible publications of types not normally indexed in electronic databases were missed. Although we did extract the level of evidence associated with the recommendations we identified, the quality of the evidence varied, and was not formally assessed due to the nature of the review. Our reviews included evidence from all publication types, most of which were narrative review publications. There is no tool for assessing the quality of evidence underpinning recommendations that would be appropriate for such reviews, the majority of the publications, which could also be applied to all other included publications [47]. As in previous qualitative systematic reviews supporting reporting guideline development, we included all eligible studies regardless of their quality [47]. Quality assessment was not necessary to meet the objectives of our systematic reviews, as they were designed to gather all relevant descriptive evidence and recommendations, and to report qualitative rather than quantitative results in accordance to best practices for reporting guideline development [43]. Due to the lack of publications that focus on the reporting of pediatric trials and protocols, and the difficulty of identifying a priori which papers would contain evidence and recommendations for reporting items, our study was structured to maximize sensitivity and capture all available evidence.

Our two systematic reviews build on the SPIRIT systematic review by Tetzlaff et al. [23], but include evidence from all publication types rather than from guidelines alone, and focus on pediatrics, where a need for guidance has been identified. This approach will help to improve the legitimacy of guidelines built upon this evidence, and to contain bias that would result from creating guidelines based on expert opinion alone. Our extensive search of multiple databases without time limits, along with our sensitive approach to gathering evidence, maximized the applicable evidence that could be identified. The qualitative nature of the data extraction process ensured that the context of the recommendations and evidence were contained within the reviews. We believe this allows the results to be useable and translatable to guideline developers, informing the necessary next step in creating pediatric reporting guideline extensions. Such extensions will provide guidance to the authors of pediatric protocols, helping to alleviate concerns of incomplete protocol reporting and will answer the repeated calls for pediatric-specific trial reporting to address the common repeated deficiencies in reporting of pediatric trial reports. Together, SPIRIT-C and CONSORT-C, when adopted by authors, will ensure that pediatric research is reported in a way that ensures it is useful to all stakeholders, that bias is minimized, and that the research is not wasted.

## Conclusions

Our systematic reviews identified evidence and recommendations for the reporting of specific items in pediatric trial protocols and reports. These results provide useful and translatable evidence and recommendations to underpin the existing SPIRIT and CONSORT guidelines for reporting items when used in pediatric trials design and reporting. Also, these results provide foundations for pediatric-specific extensions to these reporting standards.

## Additional files

Additional file 1:
**SPIRIT-C search strategy for Ovid MEDLINE® (including in-process & other non-indexed citations) 1946 to July 7 2014.** (PDF 225 kb) Additional file 2:
**CONSORT-C search strategy for Ovid MEDLINE® (including in-process and other non-indexed citations) 1946 to May 16 2014.** (PDF 225 kb) Additional file 3:
**SPIRIT-C data extraction form.** (PDF 530 kb) Additional file 4:
**CONSORT-C data extraction form.** (PDF 495 kb) Additional file 5:
**Descriptive themes of evidence and recommendations identified for SPIRIT and potential SPIRIT-C extension items.** (DOCX 43 kb) Additional file 6:
**Descriptive themes of evidence and recommendations identified for CONSORT and potential CONSORT-C extension items.** (DOCX 35 kb) Additional file 7:
**Qualitative analysis: evidence from the literature organized by meaning and content, presented by descriptive themes, and sorted into analytic themes.** (PDF 191 kb)

## Abbreviations

CMR: The Cochrane Methodology Register
CONSORT: Consolidated Standards of Reporting Trials
CONSORT-C: Consolidated Standards of Reporting Trials – Children
EQUATOR: *E*nhancing the *QU*ality and *T*ransparency *O*f health *R*esearch
SPIRIT: Standard Protocol Items: Recommendations for Interventional Trials
SPIRIT-C: Standard Protocol Items: Recommendations for Interventional Trials – Children
