# Supplementary material for: Recommendations and evidence for reporting items in pediatric clinical trial protocols and reports: two systematic reviews
Source: Trials. 2015 Sep 18;16:417. doi: 10.1186/s13063-015-0954-0 (PMC4574457; doi:10.1186/s13063-015-0954-0)
Supplement: Additional file 1: — SPIRIT-C search strategy for Ovid MEDLINE® (including in-process & other non-indexed citations) 1946 to July 7 2014. (PDF 225 kb) [file 13063_2015_954_MOESM1_ESM.pdf]

SPIRIT-C search strategy for Ovid MEDLINE® (including in-process & other non-indexed citations) 1946 to July 7 2014

1. research design/ or clinical trial as topic/st, mt or biomedical research/ or writing/ or publishing/st or clinical protocols/st, mt or evidence-based practice/st or peer review, research/mt, st or documentation/mt, st

2. (child$ or paediatric$ or pediatric$ or infan$ or young$ or toddler or bab$ or preschool$ or pre-school or adolesc$ or teenage$ or youth$ or neonat$).mp.

3. (protocol$ or design$).ti,ab,mt.

4. "Standard Protocol Items: Recommendations for Interventional Trials".tw.

5. (report$ or guideline$ or checklist$ or recommend$ or standard$ or require$ or instruct$ or guidance$ or consensus or aide memoir$ or writ$).tw.

6. animal/ not human/

7. (1 and 2 and 3 and 5) or 4

8. 7 not 6

9. limit 8 to english language
