# Supplementary material for: Recommendations and evidence for reporting items in pediatric clinical trial protocols and reports: two systematic reviews
Source: Trials. 2015 Sep 18;16:417. doi: 10.1186/s13063-015-0954-0 (PMC4574457; doi:10.1186/s13063-015-0954-0)
Supplement: Additional file 2: — CONSORT-C search strategy for Ovid MEDLINE® (including in-process and other non-indexed citations) 1946 to May 16 2014. (PDF 225 kb) [file 13063_2015_954_MOESM2_ESM.pdf]

CONSORT-C search strategy for Ovid MEDLINE® (including in-process & other non-indexed citations) 1946 to May 16 2014

1. clinical trial/ or clinical trial, phase i/ or clinical trial, phase ii/ or clinical trial, phase iii/ or clinical trial, phase iv/ or controlled clinical trial/ or multicenter study/ or randomized controlled trial/ or pragmatic clinical trial/ or clinical trials as topic/ or clinical trials, phase i as topic/ or clinical trials, phase ii as topic/ or clinical trials, phase iii as topic/ or clinical trials, phase iv as topic/ or controlled clinical trials as topic/ or randomized controlled trials as topic/

2. research design/ or biomedical research/ or writing/ or publishing/ or evidence-based practice/st or peer review, research/mt, st or documentation/mt, st

3. Publishing/

4. (report$ or guideline$ or checklist$ or recommend$ or standard$ or require$ or instruct$ or guidance$ or consensus or aide memoir$ or writ$ or consort).tw.

5. (manuscript? adj3 (submit$ or submission? or writing or quality or write? or written)).tw.

6. 1 and 2 and (3 or 4 or 5)

7. (child$ or paediatric$ or pediatric$ or infan$ or young$ or toddler or bab$ or preschool$ or pre-school or adolesc$ or teenage$ or youth$ or neonat$).mp.

8. 6 and 7

9. animal/ not human/

10. 8 not 9
