# Supplementary material for: Recommendations and evidence for reporting items in pediatric clinical trial protocols and reports: two systematic reviews
Source: Trials. 2015 Sep 18;16:417. doi: 10.1186/s13063-015-0954-0 (PMC4574457; doi:10.1186/s13063-015-0954-0)
Supplement: Additional file 3: — SPIRIT-C data extraction form. (PDF 530 kb) [file 13063_2015_954_MOESM3_ESM.pdf]

SPIRIT-C data extraction form

Item	Description
Record ID	
First Author	*Last Name, First Initial (e.g. Smith, J)*
Year	
Title	
Journal	*Full title of journal*
<u>Study Information</u>	
Study Type	*1 = clinical trial*<br>*2 = systematic review*<br>*3 = meta analysis*<br>*4 = commentary/review*<br>*5 = cohort/cross sectional*<br>*6 = protocol*<br>*7 = other*
Country	
Target Population	*1 = adult*<br>*2 = paediatric*<br>*3 = all*<br>*4 = N/A*
Trial Design	*If it's a CT*<br>*1 = RCT*<br>*2 = Cluster*<br>*3 = Other*
Area of study	*e.g. Rheumatology*
<u>Guideline Details</u>	
Does this paper describe a protocol guideline/recommendations for trials?	*0 = No*<br>*1 = Yes*<br>*2 = Don't know*<br>*6 = N/A*
If the question above is yes: What study design are these guidelines intended for?	*1 = clinical trial*<br>*2 = systematic review*<br>*3 = meta analysis*<br>*4 = commentary/review*<br>*5 = cohort/cross sectional*<br>*6 = protocol*<br>*7 = other*<br><br>*NOTE: If there is more than one applicable, separate each number with ",[space]"*
Is evidence provided to support these guidelines/suggestions?	*0 = No*<br>*1 = Yes*<br>*2 = Don't know*<br>*6 = N/A*
If yes, how was this evidence achieved?	*1 = Literature Review*<br>*2 = Systematic REview*<br>*3 = Clinical Trial*<br>*4 = Consensus*<br>*5 = Expert opinion*<br>*6 = N/A*

SPIRIT Statement Evidence	
Administrative information	
Title	
1 Descriptive title identifying the study design, population, interventions, and, if applicable, trial acronym	*0 = No*<br>*1 = Yes*<br>*2 = Don't know*<br>*6 = N/A*
	Evidence
Trial registration	
2a Trial identifier and registry name. If not yet registered, name of intended registry	*0 = No*<br>*1 = Yes*<br>*2 = Don't know*<br>*6 = N/A*
	Evidence
2b All items from the World Health Organization Trial Registration Data Set (Appendix Table, available at www.annals.org)	*0 = No*<br>*1 = Yes*<br>*2 = Don't know*<br>*6 = N/A*
	Evidence
Protocol version	
3 Date and version identifier	*0 = No*<br>*1 = Yes*<br>*2 = Don't know*<br>*6 = N/A*
	Evidence
Funding	
4 Sources and types of financial, material, and other support	*0 = No*<br>*1 = Yes*<br>*2 = Don't know*<br>*6 = N/A*
	Evidence
Roles and responsibilities	
5a Names, affiliations, and roles of protocol contributors	*0 = No*<br>*1 = Yes*<br>*2 = Don't know*<br>*6 = N/A*
	Evidence
5b Name and contact information for the trial sponsor	*0 = No*<br>*1 = Yes*<br>*2 = Don't know*<br>*6 = N/A*
	Evidence
5c Role of study sponsor and funders, if any, in study design; collection, management, analysis, and interpretation of data; writing of the report; and the decision to submit the report for publication, including whether they will have ultimate authority over any of these activities	*0 = No*<br>*1 = Yes*<br>*2 = Don't know*<br>*6 = N/A*
	Evidence
5d Composition, roles, and responsibilities of the coordinating center, steering committee, end point adjudication committee, data management team, and other individuals or groups overseeing the trial, if applicable (see item 21a for DMC)	*0 = No*<br>*1 = Yes*<br>*2 = Don't know*<br>*6 = N/A*
	Evidence
Introduction	

Background and rationale	
6a Description of research question and justification for undertaking the trial, including summary of relevant studies (published and unpublished) examining benefits and harms for each intervention	*0 = No*<br>*1 = Yes*<br>*2 = Don't know*<br>*6 = N/A*<br>*Evidence*
6b Explanation for choice of comparators	*0 = No*<br>*1 = Yes*<br>*2 = Don't know*<br>*6 = N/A*<br>*Evidence*
Objectives	
7 Specific objectives or hypotheses	*0 = No*<br>*1 = Yes*<br>*2 = Don't know*<br>*6 = N/A*<br>*Evidence*
Trial design	
8 Description of trial design, including type of trial (e.g., parallel group, crossover, factorial, single group), allocation ratio, and framework (e.g., superiority, equivalence, noninferiority, exploratory)	*0 = No*<br>*1 = Yes*<br>*2 = Don't know*<br>*6 = N/A*<br>*Evidence*
Methods	
Participants, interventions, and outcomes	
Study setting	
9 Description of study settings (e.g., community clinic, academic hospital) and list of countries where data will be collected. Reference to where list of study sites can be obtained	*0 = No*<br>*1 = Yes*<br>*2 = Don't know*<br>*6 = N/A*<br>*Evidence*
Eligibility criteria	
10 Inclusion and exclusion criteria for participants. If applicable, eligibility criteria for study centers and individuals who will perform the interventions (e.g., surgeons, psychotherapists)	*0 = No*<br>*1 = Yes*<br>*2 = Don't know*<br>*6 = N/A*<br>*Evidence*
Interventions	
11a Interventions for each group with sufficient detail to allow replication, including how and when they will be administered	*0 = No*<br>*1 = Yes*<br>*2 = Don't know*<br>*6 = N/A*<br>*Evidence*
11b Criteria for discontinuing or modifying allocated interventions for a given trial participant (e.g., drug dose change in response to harms, participant request, or improving/worsening disease)	*0 = No*<br>*1 = Yes*<br>*2 = Don't know*<br>*6 = N/A*<br>*Evidence*
11c Strategies to improve adherence to intervention protocols, and any procedures for monitoring adherence (e.g., drug tablet return, laboratory tests)	*0 = No*<br>*1 = Yes*<br>*2 = Don't know*<br>*6 = N/A*<br>*Evidence*

11d Relevant concomitant care and interventions that are permitted or prohibited during the trial	*0 = No*<br>*1 = Yes*<br>*2 = Don't know*<br>*6 = N/A*<br>*Evidence*
Outcomes	
12 Primary, secondary, and other outcomes, including the specific measurement variable (e.g., systolic blood pressure), analysis metric (e.g., change from baseline, final value, time to event), method of aggregation (e.g., median, proportion), and time point for each outcome. Explanation of the clinical relevance of chosen efficacy and harm outcomes is strongly recommended	*0 = No*<br>*1 = Yes*<br>*2 = Don't know*<br>*6 = N/A*<br>*Evidence*
Participant timeline	
13 Time schedule of enrollment, interventions (including any run-ins and washouts), assessments, and visits for participants. A schematic diagram is highly recommended	*0 = No*<br>*1 = Yes*<br>*2 = Don't know*<br>*6 = N/A*<br>*Evidence*
Sample size	
14 Estimated number of participants needed to achieve study objectives and how it was determined, including clinical and statistical assumptions supporting any sample size calculations	*0 = No*<br>*1 = Yes*<br>*2 = Don't know*<br>*6 = N/A*<br>*Evidence*
Recruitment	
15 Strategies for achieving adequate participant enrollment to reach target sample size	*0 = No*<br>*1 = Yes*<br>*2 = Don't know*<br>*6 = N/A*<br>*Evidence*
Assignment of interventions (for controlled trials): Allocation	
Sequence generation	
16a Method of generating the allocation sequence (e.g., computer-generated random numbers), and list of any factors for stratification. To reduce predictability of a random sequence, details of any planned restriction (e.g., blocking) should be provided in a separate document that is unavailable to those who enroll participants or assign interventions.	*0 = No*<br>*1 = Yes*<br>*2 = Don't know*<br>*6 = N/A*<br>*Evidence*
Allocation concealment mechanism	
16b Mechanism of implementing the allocation sequence (e.g., central telephone; sequentially numbered, opaque, sealed envelopes), describing any steps to conceal the sequence until interventions are assigned	*0 = No*<br>*1 = Yes*<br>*2 = Don't know*<br>*6 = N/A*<br>*Evidence*
Implementation	
16c Who will generate the allocation sequence, who will enroll participants, and who will assign participants to interventions	*0 = No*<br>*1 = Yes*<br>*2 = Don't know*<br>*6 = N/A*<br>*Evidence*
Blinding (masking)	
17a Who will be blinded after assignment to interventions (e.g., trial participants, care providers, outcome assessors, data analysts), and how	*0 = No*<br>*1 = Yes*<br>*2 = Don't know*<br>*6 = N/A*

	Evidence
17b If blinded, circumstances under which unblinding is permissible, and procedure for revealing a participant's allocated intervention during the trial	*0 = No*<br>*1 = Yes*<br>*2 = Don't know*<br>*6 = N/A*
	Evidence
Data collection, management, and analysis	
Data collection methods	
18a Plans for assessment and collection of outcome, baseline, and other trial data, including any related processes to promote data quality (e.g., duplicate measurements, training of assessors) and a description of study instruments (e.g., questionnaires, laboratory tests) along with their reliability and validity, if known. Reference to where data collection forms can be found, if not in the protocol.	*0 = No*<br>*1 = Yes*<br>*2 = Don't know*<br>*6 = N/A*
	Evidence
18b Plans to promote participant retention and complete follow-up, including list of any outcome data to be collected for participants who discontinue or deviate from intervention protocols	*0 = No*<br>*1 = Yes*<br>*2 = Don't know*<br>*6 = N/A*
	Evidence
Data management	
19 Plans for data entry, coding, security, and storage, including any related processes to promote data quality (e.g., double data entry; range checks for data values). Reference to where details of data management procedures can be found, if not in the protocol.	*0 = No*<br>*1 = Yes*<br>*2 = Don't know*<br>*6 = N/A*
	Evidence
Statistical methods	
20a Statistical methods for analyzing primary and secondary outcomes. Reference to where other details of the statistical analysis plan can be found, if not in the protocol.	*0 = No*<br>*1 = Yes*<br>*2 = Don't know*<br>*6 = N/A*
	Evidence
20b Methods for any additional analyses (e.g., subgroup and adjusted analyses)	*0 = No*<br>*1 = Yes*<br>*2 = Don't know*<br>*6 = N/A*
	Evidence
20c Definition of analysis population relating to protocol nonadherence (e.g., as-randomized analysis), and any statistical methods to handle missing data (e.g., multiple imputation)	*0 = No*<br>*1 = Yes*<br>*2 = Don't know*<br>*6 = N/A*
	Evidence
Monitoring	
Data monitoring	
21a Composition of DMC; summary of its role and reporting structure; statement of whether it is independent from the sponsor and competing interests; and reference to where further details about its charter can be found, if not in the protocol. Alternatively, an explanation of why a DMC is not needed.	*0 = No*<br>*1 = Yes*<br>*2 = Don't know*<br>*6 = N/A*
	Evidence

21b Description of any interim analyses and stopping guidelines, including who will have access to these interim results and<br>make the final decision to terminate the trial	*0 = No*<br>*1 = Yes*<br>*2 = Don't know*<br>*6 = N/A*
	Evidence
Harms	
22 Plans for collecting, assessing, reporting, and managing solicited and spontaneously reported adverse events and other unintended effects of trial interventions or trial conduct	*0 = No*<br>*1 = Yes*<br>*2 = Don't know*<br>*6 = N/A*
	Evidence
Auditing	
23 Frequency and procedures for auditing trial conduct, if any, and whether the process will be independent from<br>investigators and the sponsor	*0 = No*<br>*1 = Yes*<br>*2 = Don't know*<br>*6 = N/A*
	Evidence
Ethics and dissemination	
Research ethics approval	
24 Plans for seeking REC/IRB approval	*0 = No*<br>*1 = Yes*<br>*2 = Don't know*<br>*6 = N/A*
	Evidence
Protocol amendments	
25 Plans for communicating important protocol modifications (e.g., changes to eligibility criteria, outcomes, analyses) to<br>relevant parties (e.g., investigators, RECs/IRBs, trial participants, trial registries, journals, regulators)	*0 = No*<br>*1 = Yes*<br>*2 = Don't know*<br>*6 = N/A*
	Evidence
Consent or assent	
26a Who will obtain informed consent or assent from potential trial participants or authorized surrogates, and how (see item 32)	*0 = No*<br>*1 = Yes*<br>*2 = Don't know*<br>*6 = N/A*
	Evidence
26b Additional consent provisions for collection and use of participant data and biological specimens in ancillary studies, if applicable	*0 = No*<br>*1 = Yes*<br>*2 = Don't know*<br>*6 = N/A*
	Evidence
Confidentiality	
27 How personal information about potential and enrolled participants will be collected, shared, and maintained in order to protect confidentiality before, during, and after the trial	*0 = No*<br>*1 = Yes*<br>*2 = Don't know*<br>*6 = N/A*
	Evidence
Declaration of interests	
28 Financial and other competing interests for principal investigators for the overall trial and each study site	*0 = No*<br>*1 = Yes*<br>*2 = Don't know*<br>*6 = N/A*
	Evidence

Access to data	
29 Statement of who will have access to the final trial data set, and disclosure of contractual agreements that limit such access for investigators	*0 = No*<br>*1 = Yes*<br>*2 = Don't know*<br>*6 = N/A*<br>*Evidence*
Ancillary and post-trial care	
30 Provisions, if any, for ancillary and post-trial care, and for compensation to those who suffer harm from trial participation	*0 = No*<br>*1 = Yes*<br>*2 = Don't know*<br>*6 = N/A*<br>*Evidence*
Dissemination policy	
31a Plans for investigators and sponsor to communicate trial results to participants, health care professionals, the public, and other relevant groups (e.g., via publication, reporting in results databases, or other data-sharing arrangements), including any publication restrictions	*0 = No*<br>*1 = Yes*<br>*2 = Don't know*<br>*6 = N/A*<br>*Evidence*
31b Authorship eligibility guidelines and any intended use of professional writers	*0 = No*<br>*1 = Yes*<br>*2 = Don't know*<br>*6 = N/A*<br>*Evidence*
31c Plans, if any, for granting public access to the full protocol, participant-level data set, and statistical code	*0 = No*<br>*1 = Yes*<br>*2 = Don't know*<br>*6 = N/A*<br>*Evidence*
Appendices	
Informed consent materials	
32 Model consent form and other related documentation given to participants and authorized surrogates	*0 = No*<br>*1 = Yes*<br>*2 = Don't know*<br>*6 = N/A*<br>*Evidence*
Biological specimens	
33 Plans for collection, laboratory evaluation, and storage of biological specimens for genetic or molecular analysis in the<br>current trial and for future use in ancillary studies, if applicable	*0 = No*<br>*1 = Yes*<br>*2 = Don't know*<br>*6 = N/A*<br>*Evidence*
SPIRIT-C Evidence	
Administration information	
Title	
1b. Title identifying a pediatric clinical trial, with an indication of the ages group(s)	*0 = No*<br>*1 = Yes*<br>*2 = Don't know*<br>*6 = N/A*<br>*Evidence*
Roles and responsibilities	

5d3. Detail of roles and responsibilities of Data Monitoring Committees	*0 = No*<br>*1 = Yes*<br>*2 = Don't know*<br>*6 = N/A*
	Evidence
Introduction	
Background and rationale	
6a2. Identification or completion of a systematic review of all previous studies	*0 = No*<br>*1 = Yes*<br>*2 = Don't know*<br>*6 = N/A*
	Evidence
6a3. Description of potential for extrapolation from available adult data<br><br>6a4. Description of why extrapolation is not considered possible and an interventional study is considered necessary	*0 = No*<br>*1 = Yes*<br>*2 = Don't know*<br>*6 = N/A*
	Evidence
6b2. Justification of the use of comparators in relation to the pediatric population in terms of scientific and ethical implications	*0 = No*<br>*1 = Yes*<br>*2 = Don't know*<br>*6 = N/A*
	Evidence
Methods	
Participants, interventions, and outcomes: study setting	
9d. Description of efforts to reduce risk of participation	*0 = No*<br>*1 = Yes*<br>*2 = Don't know*<br>*6 = N/A*
	Evidence
Eligibility criteria	
10b. Justification of the age group selected to investigate the treatment effect and explanation of the age-related differences (biological, developmental, psychological and social) in the treatment effect of an intervention<br><br>10c. Rationale for sub-grouping of the study population in investigating the treatment effect and justify the choice of age groups/sub-grouping based on age in respect to a particular subspecialty, trial topic and/or intervention	*0 = No*<br>*1 = Yes*<br>*2 = Don't know*<br>*6 = N/A*
	Evidence
10d. Justification for the diagnostic maneuver / biomarkers used to select pediatric patients for inclusion or exclusion<br><br>10f. Justification of all diagnostic tests or evaluations to establish eligibility	*0 = No*<br>*1 = Yes*<br>*2 = Don't know*<br>*6 = N/A*
	Evidence
10e. Pre-specify the baselines variables that will be assessed in each age group and if possible describe their effect on the stated primary outcome	*0 = No*<br>*1 = Yes*<br>*2 = Don't know*<br>*6 = N/A*
	Evidence
Interventions	
11a2. Justification of the suitability of the chosen interventions to the pediatric population and to the pre-specified sub-groups in terms of the dose, duration, strength, route of administration, bioavailability, manipulation of adult dose for each intervention and treatment fidelity in case of behavioral trials	*0 = No*<br>*1 = Yes*<br>*2 = Don't know*<br>*6 = N/A*

	Evidence
11e. Description of the appropriateness of the following processes to the pediatric population and pre-specified sub-groups: Standard criteria for intervention modification and discontinuation	*0 = No*<br>*1 = Yes*<br>*2 = Don't know*<br>*6 = N/A*
	Evidence
Outcomes	
12b. Explanation of the relevance of the selected outcomes (benefits and harms) to the pediatric population and to the pre-specified age group(s) in terms of differences in disease definition pathogenesis, physiology, pharmacology), different clinical features and natural history, clinical practice, and roles within the contexts of families and society in general	*0 = No*<br>*1 = Yes*<br>*2 = Don't know*<br>*6 = N/A*
	Evidence
12d. Description of measurement properties of the instruments/scales used to measure the selected outcomes, especially those related to their responsiveness to change<br><br>12g. Description of who is measuring each of the primary and secondary outcomes, and adverse events (e.g. child, care provider, investigator etc.)	*0 = No*<br>*1 = Yes*<br>*2 = Don't know*<br>*6 = N/A*
	Evidence
12e. Description of potential short term harms<br><br>12f. Description of how long term safety is addressed	*0 = No*<br>*1 = Yes*<br>*2 = Don't know*<br>*6 = N/A*
	Evidence
Assignment of interventions (for controlled trials): Allocation	
Blinding (masking)	
17a2. Statement indicating whether children and their care-givers will be blinded to the intervention	*0 = No*<br>*1 = Yes*<br>*2 = Don't know*<br>*6 = N/A*
	Evidence
Monitoring: Harms	
22b. Explanation of the relevance of anticipated harms (adverse events/effects) to the pediatric population and to the pre-specified age group(s)	*0 = No*<br>*1 = Yes*<br>*2 = Don't know*<br>*6 = N/A*
	Evidence
Ethics and dissemination	
Consent or assent	
26a4. Justification for the use for proxy consent and indication of who will be eligible to provide it	*0 = No*<br>*1 = Yes*<br>*2 = Don't know*<br>*6 = N/A*
	Evidence
26b2. Indication of whether approval will be sought from local ethics committees, in case of vulnerable developing country population	*0 = No*<br>*1 = Yes*<br>*2 = Don't know*<br>*6 = N/A*
	Evidence
Declaration of interests	
28b. Financial and other competing interest for the sponsors and/or DMC members (if already identified) for the overall trial and each study site	*0 = No*<br>*1 = Yes*<br>*2 = Don't know*<br>*6 = N/A*

	Evidence
Ancillary and post-trial care	
30b. Statement indicating plans for long-term monitoring of outcomes, considering the effect of an intervention on the pediatric population and the pre-specified age group(s) beyond the formal study completion date	*0 = No*<br>*1 = Yes*<br>*2 = Don't know*<br>*6 = N/A*
	Evidence
Are there any other suggestions not included in SPIRIT-C (Original + extension)?	
Do the authors recommend the use of SPIRIT?	*0 = No*<br>*1 = Yes*<br>*2 = Don't know*
<u>Other</u>	
Does this paper describe trial methodology design issues that are specific to trials with children?	*0 = No*<br>*1 = Yes*<br>*2 = Don't know*
Were there any methodological design issues addressed with respect to children that (might) deviate from adults?	*0 = No*<br>*1 = Yes*<br>*2 = Don't know*
If yes, list the issues	*Copy the specific section into a separate sheet in this workbook*
Describe how these issues may have been overcome	*Copy the specific section into a separate sheet in this workbook*
Does this paper describe ethical issues that are specific to the design of trials with children?	*0 = No*<br>*1 = Yes*<br>*2 = Don't know*
If yes, list the issues	*Copy the specific section into a separate sheet in this workbook*
Describe how these issues may have been overcome	*Copy the specific section into a separate sheet in this workbook*
Was the review of a separate paper required?	*0 = No*<br>*1 = Yes*<br>*2 = Don't know*
Cite the paper:	
Exclusion	*0 = No*<br>*1 = Yes*<br>*2 = Don't know*
If so, why?	
