# Supplementary material for: Recommendations and evidence for reporting items in pediatric clinical trial protocols and reports: two systematic reviews
Source: Trials. 2015 Sep 18;16:417. doi: 10.1186/s13063-015-0954-0 (PMC4574457; doi:10.1186/s13063-015-0954-0)
Supplement: Additional file 4: — CONSORT-C data extraction form. (PDF 495 kb) [file 13063_2015_954_MOESM4_ESM.pdf]

CONSORT-C data extraction form

**Item**	**Description**
Record ID	
First Author	*Last Name, First Initial (e.g. Smith, J)*
Year	
Title	
Journal	*Full title of journal*
Study Information	
Study Type	*1 = clinical trial*<br>*2 = systematic review*<br>*3 = meta analysis*<br>*4 = commentary/review*<br>*5 = cohort/cross sectional*<br>*6 = protocol*<br>*7 = other*
Country	
Target Population	*1 = adult*<br>*2 = paediatric*<br>*3 = all*<br>*4 = N/A*
Trial Design	*If it's a CT*<br>*1 = RCT*<br>*2 = Cluster*<br>*3 = Other*
Area of study	*e.g. Rheumatology*
Guideline Details	
Does this paper describe a reporting guideline/recommendations for trials?	*0 = No*<br>*1 = Yes*<br>*2 = Don't know*<br>*6 = N/A*
If the question above is yes: What study design are these guidelines intended for?	*1 = clinical trial*<br>*2 = systematic review*<br>*3 = meta analysis*<br>*4 = commentary/review*<br>*5 = cohort/cross sectional*<br>*6 = protocol*<br>*7 = other*<br><br>*NOTE: If there is more than one applicable, separate each number with ",[space]"*
Is evidence provided to support these guidelines/suggestions?	*0 = No*<br>*1 = Yes*<br>*2 = Don't know*<br>*6 = N/A*
If yes, how was this evidence achieved?	*1 = Literature Review*<br>*2 = Systematic REview*<br>*3 = Clinical Trial*<br>*4 = Consensus*<br>*5 = Expert opinion*<br>*6 = N/A*
CONSORT 2010 Evidence	
Title and Abstract	
1a Identification as a randomised trial in the title	*0 = No*<br>*1 = Yes*<br>*2 = Don't know*<br>*6 = N/A*
	Evidence

1b Structured summary of trial design, method, results, and conclusions	*0 = No*<br>*1 = Yes*<br>*2 = Don't know*<br>*6 = N/A*<br>*Evidence*
<u>*Introduction*</u>	
Background & Objectives	
2a Scientific background and explanation of rationale	*0 = No*<br>*1 = Yes*<br>*2 = Don't know*<br>*6 = N/A*<br>*Evidence*
2b Specific objectives or hypotheses	*0 = No*<br>*1 = Yes*<br>*2 = Don't know*<br>*6 = N/A*<br>*Evidence*
<u>*Methods*</u>	
Trial Design	
3a Description of trial design (such as parallel, factorial) including allocation ratio	*0 = No*<br>*1 = Yes*<br>*2 = Don't know*<br>*6 = N/A*<br>*Evidence*
3b Important changes to methods after trial commencement (such as eligibility criteria), with reasons	*0 = No*<br>*1 = Yes*<br>*2 = Don't know*<br>*6 = N/A*<br>*Evidence*
Participants	
4a Eligibility criteria for participants	*0 = No*<br>*1 = Yes*<br>*2 = Don't know*<br>*6 = N/A*<br>*Evidence*
4b Settings and locations where the data were collected	*0 = No*<br>*1 = Yes*<br>*2 = Don't know*<br>*6 = N/A*<br>*Evidence*
Interventions	
5 The interventions for each group with sufficient details to allow replications, including how and when they were actually administered	*0 = No*<br>*1 = Yes*<br>*2 = Don't know*<br>*6 = N/A*<br>*Evidence*
Outcomes	
6a Completely defined pre-specified primary and secondary outcome measures, including how and when they were assessed	*0 = No*<br>*1 = Yes*<br>*2 = Don't know*<br>*6 = N/A*<br>*Evidence*
6b Any changes to trial outcomes after the trial commenced, with reasons	*0 = No*<br>*1 = Yes*<br>*2 = Don't know*<br>*6 = N/A*<br>*Evidence*
Sample Size	

7a How sample size was determined	*0 = No*<br>*1 = Yes*<br>*2 = Don't know*<br>*6 = N/A*
	Evidence
7b When applicable, explanation of any interim anaylses and stopping guidelines	*0 = No*<br>*1 = Yes*<br>*2 = Don't know*<br>*6 = N/A*
	Evidence
Randomization	
Sequence Generation	
8a Method used to generate the random allocation sequence	*0 = No*<br>*1 = Yes*<br>*2 = Don't know*<br>*6 = N/A*
	Evidence
8b Type of randomisation; details of any restriction (such as blocking and block size)	*0 = No*<br>*1 = Yes*<br>*2 = Don't know*<br>*6 = N/A*
	Evidence
Allocation Concealment Mechanism	
9 Mechanism used to implement the random allocation sequence (such as sequentilly numbered containers), describing any steps taken to conceal the sequence until interventions were assigned	*0 = No*<br>*1 = Yes*<br>*2 = Don't know*<br>*6 = N/A*
	Evidence
Implementation	
10 Who generated the random allocation sequence who enrolled participants, and who assigned participants to interventions	*0 = No*<br>*1 = Yes*<br>*2 = Don't know*<br>*6 = N/A*
	Evidence
Blinding	
11a If done, who was blinded after assignment to interventions (for examples, participants, care providers, those assessing outcomes) and how	*0 = No*<br>*1 = Yes*<br>*2 = Don't know*<br>*6 = N/A*
	Evidence
11b If relevant, description of the similarity of interventions	*0 = No*<br>*1 = Yes*<br>*2 = Don't know*<br>*6 = N/A*
	Evidence
Statistical Methods	
12a Statistical methods used to compare groups for primary secondary outcomes	*0 = No*<br>*1 = Yes*<br>*2 = Don't know*<br>*6 = N/A*
	Evidence
12b Methods for additional analyses, such as subgroup analyses and adjusted analyses	*0 = No*<br>*1 = Yes*<br>*2 = Don't know*<br>*6 = N/A*
	Evidence
Results	
Participant flow	

13a For each group, the numbers of participants who were randomly assigned, received intended treatment, and were analysed for the primary outcome	*0 = No*<br>*1 = Yes*<br>*2 = Don't know*<br>*6 = N/A*<br>*Evidence*
13b For each group, losses and exclusions after randomisation, together with reasons	*0 = No*<br>*1 = Yes*<br>*2 = Don't know*<br>*6 = N/A*<br>*Evidence*
Recruitment	
14a Dates defining the periods of recruitment and follow-up	*0 = No*<br>*1 = Yes*<br>*2 = Don't know*<br>*6 = N/A*<br>*Evidence*
14b Why the trial ended or was stopped	*0 = No*<br>*1 = Yes*<br>*2 = Don't know*<br>*6 = N/A*<br>*Evidence*
Baseline data	
15 A table showing baseline demographic and clinical characteristics for each group	*0 = No*<br>*1 = Yes*<br>*2 = Don't know*<br>*6 = N/A*<br>*Evidence*
Numbers analysed	
16 For each group, number of participants (denominator) included in each analysis and whether the analysis was by original assigned groups	*0 = No*<br>*1 = Yes*<br>*2 = Don't know*<br>*6 = N/A*<br>*Evidence*
Outcomes and Estimation	
17a For each primary and secondary outcome, results for each group, and the estimated effect size and its precision (Such as 95% confidence interval)	*0 = No*<br>*1 = Yes*<br>*2 = Don't know*<br>*6 = N/A*<br>*Evidence*
17b For binary outcomes, presentation of both absolute and relative effect sizes is recommended	*0 = No*<br>*1 = Yes*<br>*2 = Don't know*<br>*6 = N/A*<br>*Evidence*
Ancillary analyses	
18 Results of any other analyses performed, including subgroup analyses and adjusted analyses, distinguishing pre-specified form exploratory	*0 = No*<br>*1 = Yes*<br>*2 = Don't know*<br>*6 = N/A*<br>*Evidence*
Harms	
19 All important harms or unintended effects in each group	*0 = No*<br>*1 = Yes*<br>*2 = Don't know*<br>*6 = N/A*<br>*Evidence*
Discussion	
Limitations	

20 Trial limitations, addressing sources of potential bias, imprecision, and, if relevant, multiplicity of analyses	*0 = No*<br>*1 = Yes*<br>*2 = Don't know*<br>*6 = N/A*
	Evidence
Generalisability	
21 Generalisability (external validity, applicability) of the trial findings	*0 = No*<br>*1 = Yes*<br>*2 = Don't know*<br>*6 = N/A*
	Evidence
Interpretation	
22 Interpretation consistent with results, balancing benefits and harms, and considering other relevant evidence	*0 = No*<br>*1 = Yes*<br>*2 = Don't know*<br>*6 = N/A*
	Evidence
Other information	
Registration	
23 Registration number and name of trial registry	*0 = No*<br>*1 = Yes*<br>*2 = Don't know*<br>*6 = N/A*
	Evidence
Protocol	
24 Where the full trial protocol can be accessed, if available	*0 = No*<br>*1 = Yes*<br>*2 = Don't know*<br>*6 = N/A*
	Evidence
Funding	
25 Sources of funding and other support (such as supply of drugs), role of funders	*0 = No*<br>*1 = Yes*<br>*2 = Don't know*<br>*6 = N/A*
	Evidence
CONSORT-C Evidence	
Title and Abstract	
Age range of participants	*0 = No*<br>*1 = Yes*<br>*2 = Don't know*<br>*6 = N/A*
	Evidence
Clearly describe the efficacy/effectiveness in children or state that there is a lack of evidence for these	*0 = No*<br>*1 = Yes*<br>*2 = Don't know*<br>*6 = N/A*
	Evidence
Introduction	
Background & Objectives	
Describe the reason to perform the clinical trial in children	*0 = No*<br>*1 = Yes*<br>*2 = Don't know*<br>*6 = N/A*
	Evidence

Were parents and children involved in planning the trial?	*0 = No*<br>*1 = Yes*<br>*2 = Don't know*<br>*6 = N/A*
	Evidence
Report whethere there is a systematic review of the intervention and whether it includes children	*0 = No*<br>*1 = Yes*<br>*2 = Don't know*<br>*6 = N/A*
	Evidence
For drug studies, describe what is known about the differences in pharmacokinetics and pharmacodynamics between children and adults	*0 = No*<br>*1 = Yes*<br>*2 = Don't know*<br>*6 = N/A*
	Evidence
Methods	
Trial Design	
Report whether a Data Safety Monitoring Board (or Data Monitoring Committee) was established	*0 = No*<br>*1 = Yes*<br>*2 = Don't know*<br>*6 = N/A*
	Evidence
Participants	
Specific the age range for eligible children	*0 = No*<br>*1 = Yes*<br>*2 = Don't know*<br>*6 = N/A*
	Evidence
Rationale for the age range(s) selected for the trial	*0 = No*<br>*1 = Yes*<br>*2 = Don't know*<br>*6 = N/A*
	Evidence
Intervention	
Dose form, strength of formulation used, bioavailability, excipients, rationale for choice, manipulation of adult dose	*0 = No*<br>*1 = Yes*<br>*2 = Don't know*<br>*6 = N/A*
	Evidence
Rationale and level of evidence for control (active comparator)	*0 = No*<br>*1 = Yes*<br>*2 = Don't know*<br>*6 = N/A*
	Evidence
How much blood was drawn for the purpose of research over the course of the study?	*0 = No*<br>*1 = Yes*<br>*2 = Don't know*<br>*6 = N/A*
	Evidence
Outcomes	
Validity of outcomes in age group(s) included	*0 = No*<br>*1 = Yes*<br>*2 = Don't know*<br>*6 = N/A*
	Evidence
Sample Size	
Implications of planned subgroup analyses on sample size	*0 = No*<br>*1 = Yes*<br>*2 = Don't know*<br>*6 = N/A*
	Evidence

<u>Randomization:</u>	
Sequence Generation	
Was stratified randomization considered?	*0 = No*<br>*1 = Yes*<br>*2 = Don't know*<br>*6 = N/A*<br>*Evidence*
Statistical Methods	
Was effect modification by age, sex, anthropometric status, and (if relevant to age of particiapnts) gestation, birthweight, and breastfeeding status considered?	*0 = No*<br>*1 = Yes*<br>*2 = Don't know*<br>*6 = N/A*<br>*Evidence*
Ethical Considerations	
Was information about research provided to children and assent taken (appropriate for age)?	*0 = No*<br>*1 = Yes*<br>*2 = Don't know*<br>*6 = N/A*<br>*Evidence*
What measures were taken to reduce pain, distress, and invasiveness of research methods?	*0 = No*<br>*1 = Yes*<br>*2 = Don't know*<br>*6 = N/A*<br>*Evidence*
<u>Results</u>	
Baseline Data	
Age distribution of children in the trial	*0 = No*<br>*1 = Yes*<br>*2 = Don't know*<br>*6 = N/A*<br>*Evidence*
Number of children in the trial by age categories (0-28 days, 1-12 months, 1-2 years, 3-5 years, 5-11 years, 12-17 years)	*0 = No*<br>*1 = Yes*<br>*2 = Don't know*<br>*6 = N/A*<br>*Evidence*
Distribution by sex, nutritional status, if relevant by gestation, birthweight, breastfeeding status, pubertal stages	*0 = No*<br>*1 = Yes*<br>*2 = Don't know*<br>*6 = N/A*<br>*Evidence*
Harms	
Results of plan for a long-term adverse reactions, particularly those related to growth and development. If not, rationale for why not	*0 = No*<br>*1 = Yes*<br>*2 = Don't know*<br>*6 = N/A*<br>*Evidence*
<u>Discussion</u>	
Limitations	
Using additional considerations	*0 = No*<br>*1 = Yes*<br>*2 = Don't know*<br>*6 = N/A*<br>*Evidence*
Are there any other suggestions not included in CONSORT-C (Original + extension)?	
<u>Other</u>	

Does this paper describe trial methodology issues that are specific to trials with children?	*0 = No*<br>*1 = Yes*<br>*2 = Don't know*
Were there any methodological issues addressed with respect to children that (might) deviate from adults?	*0 = No*<br>*1 = Yes*<br>*2 = Don't know*
If yes, list the issues	*Copy the specific section into a separate sheet in this workbook*
Describe how these issues may have been overcome	*Copy the specific section into a separate sheet in this workbook*
Does this paper describe ethical issues that are specific to trials with children?	*0 = No*<br>*1 = Yes*<br>*2 = Don't know*
If yes, list the issues	*Copy the specific section into a separate sheet in this workbook*
Describe how these issues may have been overcome	*Copy the specific section into a separate sheet in this workbook*
Was the review of a separate paper required?	*0 = No*<br>*1 = Yes*<br>*2 = Don't know*
Cite the paper:	
Exclusion	*0 = No*<br>*1 = Yes*<br>*2 = Don't know*
If so, why?	
