# Supplementary material for: Recommendations and evidence for reporting items in pediatric clinical trial protocols and reports: two systematic reviews
Source: Trials. 2015 Sep 18;16:417. doi: 10.1186/s13063-015-0954-0 (PMC4574457; doi:10.1186/s13063-015-0954-0)
Supplement: Additional file 5: — Descriptive themes of evidence and recommendations identified for SPIRIT and potential SPIRIT-C extension items. (DOCX 43 kb) [file 13063_2015_954_MOESM5_ESM.docx]

| Section | Reporting Item No. | Standard SPIRIT Items | Proposed SPIRIT-C Items | Themes of Evidence  (numbers in parentheses represent number of occurrences of these themes of evidence found) | Number of publication(s) that contain recommendations for the reporting of the item | Number of publication(s) that contain evidence for the reporting of the item |
| --- | --- | --- | --- | --- | --- | --- |
| Administrative information | | | |  |  |  |
| Title | 1 | Descriptive title identifying the study design, population, interventions, and, if applicable, trial acronym |  | Helps literature searches / Helps reader evaluate relevance (10) | 5 | 1 |
|  | 1.1 |  | Title identifying a pediatric clinical trial, with an indication of the age group(s) | - | 0 | 0 |
| Trial registration | 2a | Trial identifier and registry name. If not yet registered, name of intended registry |  | Prevents reporting bias / selective reporting / publication bias (13); Public accountability / transparency / public accessibility / recruitment (4); Reduces research waste / unnecessary duplication of research / allows gaps in knowledge to be identified / Makes publications database searchable and findable (5) | 2 | 9 |
|  | 2b | All items from the World Health Organization Trial Registration Data Set |  | Structured summary / consistency of information (2) | 1 | 1 |
| Protocol version | 3 | Date and version identifier |  | Transparency / allows oversight and review / clarity / avoids confusion of which version is most recent (5) | 2 | 1 |
| Funding | 4 | Sources and types of financial, material, and other support |  | Assess potential competing interests / transparency / assess whether reimbursement amount is reasonable / risk of bias (reporting / publication bias) / assess study feasibility (12) | 6 | 2 |
| Roles and responsibilities | 5a | Names, affiliations, and roles of protocol contributors |  | Transparency / assess competing interests (11); Assess whether families, patients, or children were protocol contributors (16); Recognition / reducing ghost authorship / accountability / appropriateness of those selected for their roles, whether statisticians were included (13) | 5 | 13 |
|  | 5b | Name and contact information for the trial sponsor |  | Transparency / accountability (10) | 6 | 1 |
|  | 5c | Role of study sponsor and funders, if any, in study design; collection, management, analysis, and interpretation of data; writing of the report; and the decision to submit the report for publication, including whether they will have ultimate authority over any of these activities |  | Assess potential for bias through competing interests or influence (7) | 3 | 3 |
|  | 5d | Composition, roles, and responsibilities of the coordinating centre, steering committee, endpoint adjudication committee, data management team, and other individuals or groups overseeing the trial, if applicable (see Item 21a for data monitoring committee) |  | Clarity of roles and responsibilities (5); Assess the expertise and mandate of those responsible (4) | 2 | 2 |
|  | 5d.1 |  | Details of roles and responsibilities of Data Monitoring Committees | Assess the appropriateness of the composition (5); Assess the quality and safety assurance roles (12) | 3 | 11 |
| Introduction | | | |  |  |  |
| Background and rationale | 6a | Description of research question and justification for undertaking the trial, including summary of relevant studies (published and unpublished) examining benefits and harms for each intervention |  | Allows participants and personnel to assess the motivation for the trial (2); Allows funders and IRBs to assess the scientific and ethical justification / reduces research waste or unnecessary research / to include pediatrics requires justification (52) | 14 | 29 |
|  | 6a.1 |  | Identification or completion of a systematic review of all previous studies | Showing uncertainty or gaps/ equipoise/ justification of trial (5); Consistency with or building on prior research / ensuring the trial is useful (4) | 5 | 3 |
|  | 6a.2 |  | Description of potential for extrapolation from available adult data; Description of why extrapolation is not considered possible and an interventional study is considered necessary | Ethics / the need to extrapolate from existing data (10); Differences / the limits to extrapolation from existing data (19) | 1 | 25 |
|  | 6b | Explanation for choice of comparators |  | Allows the assessment of the scientific appropriateness of the comparators (34); Allows the assessment of the ethical appropriateness of the comparators / pediatric inability at consent to placebo risk (17); Allows clarity of "standard care" in pediatrics (11) | 2 | 40 |
|  | 6b.1 |  | Justification of the use of comparators in relation to the pediatric population in terms of scientific and ethical implications | Ethics / risks (14); Scientific implications (13); Differences (11) | 2 | 29 |
| Objectives | 7 | Specific objectives or hypotheses |  | Allows the purpose /scope of the trial to be assessed (30); Assess the appropriateness of sample size calculation, trial design, and statistical analyses (31) | 16 | 9 |
| Trial design | 8 | Description of trial design including type of trial (eg, parallel group, crossover, factorial, single group), allocation ratio, and framework (eg, superiority, equivalence, noninferiority, exploratory) |  | Assess compatibility with objectives and hypotheses / prevents post hoc equivalence claims / assess ability to use results (meta-analysis) (37) | 5 | 17 |
| Methods Participants, interventions, and outcomes | | | |  |  |  |
| Study setting | 9 | Description of study settings (eg, community clinic, academic hospital) and list of countries where data will be collected. Reference to where list of study sites can be obtained |  | Context / applicability / generalizability (23) | 12 | 15 |
|  | 9.1 |  | Description of efforts to reduce risk of participation | Pediatric specific pain/ duress/ burden / risks specific to pediatrics / off-label drug use / development risks (22); Ethics / assess the appropriateness of risk reduction / ensure they match what is done in the trial (17) | 1 | 30 |
| Eligibility criteria | 10 | Inclusion and exclusion criteria for participants. If applicable, eligibility criteria for study centres and individuals who will perform the interventions (eg, surgeons, psychotherapists) |  | Consistency of criteria between study personnel / clarity / pediatric issues - criteria may be different for pediatric populations (40); Recruitment and attrition / feasibility (27); Generalizability / applicability / reasonability of criteria (not too narrow, meets objectives) / research waste from narrow criteria (70); Assessment of differences in trial report (23) | 17 | 50 |
|  | 10.1 |  | Justification of the age group selected to investigate the treatment effect and explanation of the age-related differences (biological, developmental, psychological and social) in the treatment effect of an intervention; Rationale for sub-grouping of the study population in investigating the treatment effect and justify the choice of age groups/sub-grouping based on age in respect to a particular subspecialty, trial topic and/or intervention | Age-related differences in treatment effect (12); Justification of the age group used (7); Rationale for sub-groups (14) | 5 | 20 |
|  | 10.2 |  | Justification for the diagnostic maneuver / biomarkers used to select pediatric patients for inclusion or exclusion; Justification of all diagnostic tests or evaluations to establish eligibility | Validity in pediatric population (2) | 0 | 2 |
|  | 10.3 |  | Pre-specify the baselines variables that will be assessed in each age group and if possible describe their effect on the stated primary outcome | Validity / age-baseline interactions/Age related diagnostic issues/age-related confounders (4) | 2 | 1 |
| Interventions | 11a | Interventions for each group with sufficient detail to allow replication, including how and when they will be administered |  | Clarity / transparency / allows stakeholders to reproduce, understand, and assess the intervention (45); Standard of care / usual care must be carefully defined (30) | 16 | 21 |
|  | 11a.1 |  | Justification of the suitability of the chosen interventions to the pediatric population and to the pre-specified sub-groups in terms of the dose, duration, strength, route of administration, bioavailability, manipulation of adult dose for each intervention and treatment fidelity in case of behavioral trials | Uncertainty in pediatric pharmacokinetics / how development impacts pharmacology (7); Dose/duration/strength/route of administration (8); Differences from adults to pediatrics/ manipulation of adult dose/ limits of extrapolation (13); Differences amongst pediatric groups (5) | 1 | 22 |
|  | 11b | Criteria for discontinuing or modifying allocated interventions for a given trial participant (eg, drug dose change in response to harms, participant request, or improving/worsening disease) |  | Comparability across studies / reproducibility / objectivity improved in applying intervention (12) | 6 | 1 |
|  | 11b.1 |  | Description of the appropriateness of the following processes to the pediatric population and pre-specified sub-groups: Standard criteria for intervention modification and discontinuation | - | 0 | 0 |
|  | 11c | Strategies to improve adherence to intervention protocols, and any procedures for monitoring adherence (eg, drug tablet return, laboratory tests) |  | Adherence data informs statistical analyses / interpretation / reproducibility / implementation / issues of adherence important in pediatrics (multicenter) (29); Allows assessment of whether adherence strategy is appropriate / generalizable to real world (3) | 2 | 29 |
|  | 11d | Relevant concomitant care and interventions that are permitted or prohibited during the trial |  | Allows assessment of possible co-intervention bias / Confounding (14); Allows comparability of study groups / reproducibility / generalizability (11) | 4 | 10 |
| Outcomes | 12 | Primary, secondary, and other outcomes, including the specific measurement variable (eg, systolic blood pressure), analysis metric (eg, change from baseline, final value, time to event), method of aggregation (eg, median, proportion), and time point for each outcome. Explanation of the clinical relevance of chosen efficacy and harm outcomes is strongly recommended |  | Allows distinction of primary from other outcomes / allows assurance that same primary outcome as sample size and objective primary outcome (45); Allows assessment of possible ascertainment and selection bias, or multiplicity (15); Allows assessment of selective reporting / reporting bias (16); Allows assessment of rationale (valid, reproducible, relevant, responsive) / whether core outcome set was used / research waste, heterogeneity (40) | 12 | 60 |
|  | 12.1 |  | Explanation of the relevance of the selected outcomes (benefits and harms) to the pediatric population and to the pre-specified age group(s) in terms of differences in disease definition (pathogenesis, physiology, pharmacology), different clinical features and natural history, clinical practice, and roles within the contexts of families and society in general | Allows assessment of validity of outcomes in pediatrics versus adults (44); Validity across pediatric groups (43); Allows assessment of relevance of outcomes in pediatrics (2) | 2 | 59 |
|  | 12.2 |  | Description of measurement properties of the instruments/scales used to measure the selected outcomes, especially those related to their responsiveness to change; Description of who is measuring each of the primary and secondary outcomes, and adverse events (e.g. child, care provider, investigator etc.) | Measurement properties (validity, sensitivity, specificity, test-retest reliability) in pediatrics (24); Who measures the outcome/inter-rater variability (13) | 4 | 27 |
|  | 12.3 |  | Description of potential short term harms; Description of how long term safety is addressed | Pediatric specific harms / different short-term harms than adults (9); Long term follow-up / developmental harms (8) | 4 | 10 |
| Participant timeline | 13 | Time schedule of enrolment, interventions (including any run-ins and washouts), assessments, and visits for participants. A schematic diagram is highly recommended (see Figure) |  | Helps guide trial conduct / consistency / transparency / clarity (19); Allows external review of participant burden / feasibility (18) | 8 | 7 |
| Sample size | 14 | Estimated number of participants needed to achieve study objectives and how it was determined, including clinical and statistical assumptions supporting any sample size calculations |  | Discourages post hoc revision of calculations / Allows the critical assessment of the calculation itself, and the rationale for each assumption / component (assess for inflated clinically important effect size or underestimated standard deviation) (37); Discourages post hoc revision of margins for non-inferiority or equivalence (24); Feasibility / minimize participants to meet objectives / evaluate whether the trial will be powered to detect a difference / avoid research waste or unnecessary research / ethics (44); Ensures clustering is accounted for (21) | 10 | 38 |
| Recruitment | 15 | Strategies for achieving adequate participant enrolment to reach target sample size |  | Assess the feasibility of reaching sample size / ethics (43); Assessing generalizability / applicability / implementation to practice (12) | 4 | 40 |
| Assignment of interventions (for controlled trials) Allocation | | | |  |  |  |
| Sequence generation | 16a | Method of generating the allocation sequence (eg, computer-generated random numbers), and list of any factors for stratification. To reduce predictability of a random sequence, details of any planned restriction (eg, blocking) should be provided in a separate document that is unavailable to those who enrol participants or assign interventions |  | Assess risk of selection bias or predictability (through non-random procedures or too much information) or biased effect size (48); Allows the assessment of factors used for stratification, blocking, or minimisation for relevance (8) | 8 | 42 |
| Allocation concealment mechanism | 16b | Mechanism of implementing the allocation sequence (eg, central telephone; sequentially numbered, opaque, sealed envelopes), describing any steps to conceal the sequence until interventions are assigned |  | Assess the adequacy of the mechanism / Assess the risk of allocation concealment corruption / Assess the risk of a biased effect size (20) | 6 | 9 |
| Implementation | 16c | Who will generate the allocation sequence, who will enrol participants, and who will assign participants to interventions |  | Assess the risk of bias / risk of corruption / incomplete separation of individuals involved in steps before and after generation-concealment and implementation (14) | 6 | 3 |
| Blinding (masking) | 17a | Who will be blinded after assignment to interventions (eg, trial participants, care providers, outcome assessors, data analysts), and how |  | Assess the risk of bias / ascertainment bias / performance bias / attrition bias / risk of unblinding (49); Prevent ambiguity / who was blinded and how / how similar are interventions / reproducibility (17) | 9 | 37 |
|  | 17a.1 |  | Statement indicating whether children and their care-givers will be blinded to the intervention | - | 0 | 1 |
|  | 17b | If blinded, circumstances under which unblinding is permissible, and procedure for revealing a participant’s allocated intervention during the trial |  | Assess the appropriateness of the procedures to protect the safety of the participants / assess the appropriateness of the procedures to protect trial from unnecessary unblinding (7) | 3 | 1 |
| Data collection, management, and analysis | | | |  |  |  |
| Data collection methods | 18a | Plans for assessment and collection of outcome, baseline, and other trial data, including any related processes to promote data quality (eg, duplicate measurements, training of assessors) and a description of study instruments (eg, questionnaires, laboratory tests) along with their reliability and validity, if known. Reference to where data collection forms can be found, if not in the protocol |  | Assessment of validity & reliability of data collection methods / equal validity for both groups / validity in specific study group / risk of bias from inappropriate methods (44); Assessment of the reliability, validity, and responsiveness of instruments / Same instruments for both groups / validity in specific study group / risk of bias from inappropriate instruments (11); Assessment of processes to enhance data quality / training / duplication (17) | 7 | 45 |
|  | 18b | Plans to promote participant retention and complete follow-up, including list of any outcome data to be collected for participants who discontinue or deviate from intervention protocols |  | Assess the risk to validity and power / attrition bias (22); Assess the feasibility of the study / implementation / participant burden / ethics (8) | 3 | 21 |
| Data management | 19 | Plans for data entry, coding, security, and storage, including any related processes to promote data quality (eg, double data entry; range checks for data values). Reference to where details of data management procedures can be found, if not in the protocol |  | Assess the risk of data entry and coding issues that impact validity (2); Assess the appropriateness of procedures to promote quality / assess the pros and cons of procedures used (2); Assess the adherence to regulations / assess risks to data security / protection of data of children (2) | 0 | 3 |
| Statistical methods | 20a | Statistical methods for analysing primary and secondary outcomes. Reference to where other details of the statistical analysis plan can be found, if not in the protocol |  | Assess the risk of reporting bias / only reporting "interesting" results / assess whether the primary outcome and main comparisons remain the same in protocol and report (31); Assess the risk of multiplicity / bias of multiple tests / risk of false positive (19); Assess the appropriateness of the statistical methods, effect measure, significance level, and presentation (36) | 17 | 28 |
|  | 20b | Methods for any additional analyses (eg, subgroup and adjusted analyses) |  | Assess the risk of spurious findings in subgroup analysis / ensures post hoc analyses or categorization are identified / assess risk of multiplicity (16); Assess the appropriateness for adjusted analysis and variables used / objective criteria to select variables for adjustment / ensures it is using baseline data rather than post-randomisation data (8) | 2 | 14 |
|  | 20c | Definition of analysis population relating to protocol non-adherence (eg, as randomised analysis), and any statistical methods to handle missing data (eg, multiple imputation) |  | Assess the risk of attrition bias / selection bias by not using all randomized participants (17); Prevents ambiguity of statistical methods through labels like "intention to treat" or "per protocol" (9); Assess the appropriateness of methods of handling missing data / Assess the robustness of the assumptions made for missing data (sensitivity analysis) (14) | 2 | 16 |
| Monitoring | | | |  |  |  |
| Data monitoring | 21a | Composition of data monitoring committee (DMC); summary of its role and reporting structure; statement of whether it is independent from the sponsor and competing interests; and reference to where further details about its charter can be found, if not in the protocol. Alternatively, an explanation of why a DMC is not needed |  | Assessment of whether equipoise will be met throughout the trial & stopped when violated (16); Assessment of whether reasons of why DMC is not needed such as through short or minimally risky trials (5); Assessment of independence of DMC (6) | 7 | 15 |
|  | 21b | Description of any interim analyses and stopping guidelines, including who will have access to these interim results and make the final decision to terminate the trial |  | Assess the appropriateness of interim analyses: processes and people involved (are they blinded, are they independent, are they sponsors) / bias from influence from sponsors if they get data / assess the risk of false positives (17); Assess the appropriateness of stopping guideline: processes and people involved / assess the risk of research waste through futility or shown benefit without stopping / assess the risk of harms without stopping (22) | 6 | 9 |
| Harms | 22 | Plans for collecting, assessing, reporting, and managing solicited and spontaneously reported adverse events and other unintended effects of trial interventions or trial conduct |  | Assess the appropriateness of the monitoring for adverse events / ensure they match what is done in the trial (18); Assess the appropriateness of the reporting and management of adverse events/ ensure they match what is done in the trial (27); Assess the appropriateness of the collection procedures / ensure they match what is done in the trial (18) | 10 | 14 |
|  | 22.1 |  | Explanation of the relevance of anticipated harms (adverse events/effects) to the pediatric population and to the pre-specified age group(s) | Differences in harms across age groups (7); Risks specific to pediatrics / off-label drug use / development risks (6) | 3 | 5 |
| Auditing | 23 | Frequency and procedures for auditing trial conduct, if any, and whether the process will be independent from investigators and the sponsor |  | Assess the procedures for ensuring integrity and good clinical practice / assess the independence of the process / assess whether the trial meets regulations and policies (5) | 2 | 2 |
| Ethics and dissemination | | | |  |  |  |
| Research ethics approval | 24 | Plans for seeking research ethics committee/institutional review board (REC/IRB) approval |  | Ensures that approval is sought / a proxy for ethical evaluation of the trial (10); Pediatric necessity for this for ethics (8) | 3 | 8 |
| Protocol amendments | 25 | Plans for communicating important protocol modifications (eg, changes to eligibility criteria, outcomes, analyses) to relevant parties (eg, investigators, REC/IRBs, trial participants, trial registries, journals, regulators) |  | Assessment of whether protocol modifications will be appropriately communicated / ensures the integrity of the trial can be properly assessed / assessment of whether the relevant parties are independent (7) | 2 | 4 |
| Consent or assent | 26a | Who will obtain informed consent or assent from potential trial participants or authorised surrogates, and how (see Item 32) |  | Assess whether informed consent procedures were appropriate / lack of consent justified, if applicable (56); Assess whether proxy consent was appropriate / who decides whether proxy consent is appropriate and how (9); Assess whether informed assent procedures were appropriate / how information will be provided / how assent will be ascertained (20) | 5 | 63 |
|  | 26a.1 |  | Justification for the use for proxy consent and indication of who will be eligible to provide it | Patient competence/ maturity/understanding (29); Patient voluntariness/ assent/ dissent/ autonomy (33); Who is proxy / role of parents or guardians (45) | 4 | 63 |
|  | 26b | Additional consent provisions for collection and use of participant data and biological specimens in ancillary studies, if applicable |  | Assess whether ancillary consent procedures were appropriate (7); Assess whether the changing consent ability of children is accounted for / re-consent taken following maturity / Withdrawal if consent not given at maturity (5) | 1 | 6 |
|  | 26b.1 |  | Indication of whether approval will be sought from local ethics committees, in case of vulnerable developing country population | Standard of care/ access to intervention/ access to benefits/ no harm (3); Exploitation/ protection/ informed consent (5); Culture (2) | 0 | 5 |
| Confidentiality | 27 | How personal information about potential and enrolled participants will be collected, shared, and maintained in order to protect confidentiality before, during, and after the trial |  | Assess whether process to protect confidentiality of personal information from outsiders is appropriate (18); Assess whether confidentiality of children from parents is appropriately considered / whether personal health information is increasingly confidential with maturity (15) | 6 | 8 |
| Declaration of interests | 28 | Financial and other competing interests for principal investigators for the overall trial and each study site |  | Assess the risk of bias from competing interests / transparency (6) | 2 | 3 |
|  | 28.1 |  | Financial and other competing interests for the sponsors and/or DMC members (if already identified) for the overall trial and each study site | Independence/ conflict of interest of investigators (6); Role of sponsors (3) | 3 | 3 |
| Access to data | 29 | Statement of who will have access to the final trial dataset, and disclosure of contractual agreements that limit such access for investigators |  | Assess the ability of others to verify the validity of the trial results (5); Assess the risk of keeping results from independent validation / risk of improper procedures, too limited access / research waste / contractual agreements (7) | 1 | 5 |
| Ancillary and post-trial care | 30 | Provisions, if any, for ancillary and post-trial care, and for compensation to those who suffer harm from trial participation |  | Assess whether the researchers are fulfilling their responsibilities to participants / compensation plans (7); Assess whether interventions are available to participants following the study / if not, justification (6) | 2 | 3 |
|  | 30.1 |  | Statement indicating plans for long-term monitoring of outcomes, considering the effect of an intervention on the pediatric population and the pre-specified age group(s) beyond the formal study completion date | Effects on development (43); Rare events/ sustainability (4) | 2 | 41 |
| Dissemination policy | 31a | Plans for investigators and sponsor to communicate trial results to participants, healthcare professionals, the public, and other relevant groups (eg, via publication, reporting in results databases, or other data sharing arrangements), including any publication restrictions |  | Assess whether the ethical requirement of not wasting research is met / assess whether participants are protected from futile research (22); Assess whether the study is likely to contribute to publication bias through publication restrictions or publication delays (17) | 8 | 15 |
|  | 31b | Authorship eligibility guidelines and any intended use of professional writers |  | Assess whether bias from ghost authors will be prevented (6); Assess whether transparency and accountability is maintained, and those responsible are reachable / Assess whether guest authors will receive undue credit for work not completed (5) | 2 | 2 |
|  | 31c | Plans, if any, for granting public access to the full protocol, participant-level dataset, and statistical code |  | Assess the trial's efforts to meet ethical and scientific imperatives such as reproducible research (10) | 2 | 5 |
| Appendices | | | |  |  |  |
| Informed consent materials | 32 | Model consent form and other related documentation given to participants and authorised surrogates |  | Assess whether the consent information is appropriate for the population / age / maturity / reading level (15) | 2 | 13 |
| Biological specimens | 33 | Plans for collection, laboratory evaluation, and storage of biological specimens for genetic or molecular analysis in the current trial and for future use in ancillary studies, if applicable |  | Assess whether the processes are appropriate / assess whether pediatric specific issues (i.e. Revoking consent for storage) are met (2) | 0 | 2 |
