# Supplementary material for: Recommendations and evidence for reporting items in pediatric clinical trial protocols and reports: two systematic reviews
Source: Trials. 2015 Sep 18;16:417. doi: 10.1186/s13063-015-0954-0 (PMC4574457; doi:10.1186/s13063-015-0954-0)
Supplement: Additional file 6: — Descriptive themes of evidence and recommendations identified for CONSORT and potential CONSORT-C extension items. (DOCX 35 kb) [file 13063_2015_954_MOESM6_ESM.docx]

| Section | Reporting Item No. | Standard CONSORT Items | Proposed CONSORT-C Items | Themes of Evidence  (numbers in parentheses represent number of occurrences of these themes of evidence found) | Number of publications that contain recommendations for the reporting of the item | Number of publications that contain evidence for the reporting of the item |
| --- | --- | --- | --- | --- | --- | --- |
| Title and abstract | 1a | Identification as a randomised trial in the title |  | Helps literature searches (42); helps reader evaluate relevance (35) | 5 | 40 |
|  | 1a.1 |  | Age range of participants | - | 0 | 0 |
|  | 1b | Structured summary of trial design, methods, results, and conclusions (for specific guidance see CONSORT for abstracts) |  | Allows the reader to evaluate relevance (25); Allows the reader to make health care decisions if they do not have access to full-text (5) | 3 | 25 |
|  | 1b.1 |  | Clearly describe the efficacy/effectiveness in children or state that there is a lack of evidence for these | Evidence that there is reason to believe that the intervention could be effective / Adult data (8); The evidence is not conclusive / limits to extrapolation of efficacy from adults (10); Equipoise / lack of evidence for efficacy in pediatrics (7) | 3 | 20 |
| Introduction | | | |  |  |  |
| Background and objectives | 2a | Scientific background and explanation of rationale |  | Assess whether research participants were spared futile research / unnecessary research / ethics / can it be answered by systematic review / pediatric issues (47); Assess whether this rationale matches the methods (3) | 4 | 44 |
|  | 2a.1 |  | Describe the reason to perform the clinical trial in children | Considerations of extrapolation (14); Insufficiency of extrapolation / scientific justification (14) | 1 | 24 |
|  | 2a.2 |  | Report whether there is a systematic review of the intervention and whether it includes children | Showing uncertainty or gaps/ equipoise/justification (2) | 1 | 1 |
|  | 2a.3 |  | For drug studies, describe what is known about the differences in pharmacokinetics and pharmacodynamics between children and adults | Showing uncertainty or gaps / equipoise / justification of drug study / equipoise cannot be assumed - some drugs work the same in adults and children (17); Allowing the reader to assess the appropriateness of the dose/duration/strength/route of administration (7); Interpretation / allowing the reader to interpret differences from adults to pediatrics/ manipulation of adult dose/limits of extrapolation/ different ages (28) | 1 | 36 |
|  | 2b | Specific objectives or hypotheses |  | Allows the purpose /scope of the trial to be assessed (40); Assess the appropriateness of sample size calculation and statistical analyses (10) | 8 | 34 |
|  | 2c |  | Were parents and children involved in planning the trial? | Interpretation / allows authors to evaluate whether the interventions / outcomes were approved by parents and children (11); The authors recommend that all pediatric trials involve parents and children in planning (6); Reproducibility / allows authors to predict whether they will accrue comparable participants (6) | 0 | 17 |
| Methods | | | |  |  |  |
| Trial design | 3a | Description of trial design (such as parallel, factorial) including allocation ratio |  | Assess compatibility with objectives and hypotheses / prevents post hoc equivalence claims / ethics / clarity (27); Allow evaluation of feasibility and recruitment (1) | 1 | 27 |
|  | 3b | Important changes to methods after trial commencement (such as eligibility criteria), with reasons |  | Ensures the integrity of the trial can be properly assessed / discrepancies from protocol are explained (24); Ensures the results can be properly interpreted (3) | 1 | 23 |
|  | 3c |  | Report whether a Data Safety Monitoring Board (or Data Monitoring Committee) was established | Allows the reader to assess quality assurance / validity / appropriate stopping guidelines (14); Recommend that all pediatric trials have a DSMB (12); Allows the reader to assess safety assurance / avoidance of risks of research (13) | 4 | 21 |
| Participants | 4a | Eligibility criteria for participants |  | Reasonability of criteria (not too narrow, meets objectives) / research waste from narrow criteria (38); Allows the reader to properly interpret the results / compare the results to other studies (8); Generalizability / applicability / relevance to reader (9); Assessment of post hoc changes to eligibility criteria (3) | 4 | 38 |
|  | 4a.1 |  | Specify the age range for eligible children | Interpretation / generalizability / age-related differences in treatment effect (10); Comparison of studies / systematic review (4) | 2 | 11 |
|  | 4a.2 |  | Rationale for the age range(s) selected for the trial | Age-related differences in treatment effect (6); Justification of the age group used (2); Rationale for sub-groups / consideration for knowledge synthesis (6) | 3 | 7 |
|  | 4b | Settings and locations where the data were collected |  | Applicability / generalizability / assess whether the results apply to other settings / whether the results are relevance to the reader(38); Reasonability of chosen setting (meets objectives) / research waste from inappropriate setting chosen (6); Allows the reader to properly understand the results / compare the results to other studies (4) | 4 | 35 |
| Interventions | 5 | The interventions for each group with sufficient details to allow replication, including how and when they were actually administered |  | Allow clinicians / other researchers to use and replicate the intervention / clarity / transparency / allows stakeholders to understand, and assess the intervention (38); Standard care must be fully defined, especially in pediatrics (7) | 7 | 33 |
|  | 5.1 |  | Dose form, strength of formulation used, bioavailability, excipients, rationale for choice, manipulation of adult dose | Rationale for choice/ uncertainty in pediatric pharmacokinetics / We do not know how development impacts pharmacology (6); Allows the reader to evaluate the appropriateness of the intervention for the age / dose/duration/strength/route of administration (15); Allows the reader to evaluate whether the extrapolation was appropriate / differences from adults to pediatrics/ manipulation of adult dose/limits of extrapolation (4); Generalizability / differences amongst pediatric groups / differences between control & intervention dose (10) | 1 | 25 |
|  | 5.2 |  | Rationale and level of evidence for control (active comparator) | Ethics/ risks/ minimization of pain/ consent (21); Ability of the reader to assess the scientific validity of the trial / scientific implications (17); Differences / Ambiguousness of "normal" in children (12) | 3 | 32 |
|  | 5.3 |  | How much blood was drawn for the purpose of research over the course of the study? | Reader is able to assess whether safest procedure is used (6); Assess the ethics of the study / suffering limited / pediatric specific ethical issues (5) | 0 | 9 |
| Outcomes | 6a | Completely defined prespecified primary and secondary outcome measures, including how and when they were assessed |  | Allows others to reproduce and use the same outcomes (35); Assessment of the reliability, validity, and responsiveness of instruments / same instruments for both groups / validity in specific study group / risk of bias from inappropriate instruments (6); Allows distinction of primary from other outcomes / allows assurance that same primary outcome as sample size and objective primary outcome (8); Allows assessment of possible ascertainment and selection bias, or multiplicity (7); Allows assessment of selective reporting / reporting bias (7) | 2 | 38 |
|  | 6a.1 |  | Validity of outcomes in age group(s) included | Validity in pediatrics versus adults (71); Validity across pediatric groups (5); Measurement properties (validity, sensitivity, specificity, test-retest reliability) in pediatrics (8); Assessor of outcome/Inter-rater variability (6) | 3 | 82 |
|  | 6b | Any changes to trial outcomes after the trial commenced, with reasons |  | Ensures the integrity of the trial can be properly assessed / discrepancies from protocol are explained / avoid reporting bias and multiplicity (21); Ensures the results can be properly interpreted (5) | 3 | 21 |
| Sample size | 7a | How sample size was determined |  | Allows the critical assessment of the calculation itself, and the rationale for each assumption / component (assess for inflated clinically important effect size or underestimated standard deviation) / primary outcome was used (37); Ensures that margin for non-inferiority or equivalence were specified and justified a priori (2); Feasibility / evaluate whether the trial will be powered to detect a difference / transparency of power / allows critique of conclusions (do the groups truly not differ?) / assess whether trial was ended early (7); Ensures clustering is accounted for (4) | 6 | 33 |
|  | 7a.1 |  | Implications of planned subgroup analyses on sample size | Risk of multiplicity / spurious results (2) | 1 | 1 |
|  | 7b | When applicable, explanation of any interim analyses and stopping guidelines |  | Assess the appropriateness of interim analyses: processes and people involved (were they blinded, were they independent, who were the sponsors?) / bias from influence from sponsors if they get data / assess the risk of false positives (33); Assess the appropriateness of stopping guideline: processes and people involved / assess the risk of research waste through futility or shown benefit without stopping / assess the risk of harms without stopping (6) | 4 | 30 |
| Randomization |  |  |  |  |  |  |
| Sequence generation | 8a | Method used to generate the random allocation sequence |  | Assess the methods used / likelihood of bias in methods (38) | 2 | 36 |
|  | 8b | Type of randomisation; details of any restriction (such as blocking and block size) |  | Allows the assessment of factors used for stratification, blocking, or minimisation for relevance (35); Allows the assessment of the appropriateness of the randomization type used (2) | 3 | 32 |
|  | 8b.1 |  | Was stratified randomization considered? | Allows the evaluation of whether balance was achieved in pediatric factors (2) | 0 | 2 |
| Allocation concealment mechanism | 9 | Mechanism used to implement the random allocation sequence (such as sequentially numbered containers), describing any steps taken to conceal the sequence until interventions were assigned |  | Assess the adequacy of the mechanism / assess the risk of allocation concealment corruption / effect size bias / selection bias (35); Assures us that assignment will be truly random (12) | 3 | 34 |
| Implementation | 10 | Who generated the random allocation sequence, who enrolled participants, and who assigned participants to interventions |  | Assess whether people involved in generation and allocation concealment were separated from people involved in implementation (36); Assures us that assignment will be truly random (8) | 2 | 34 |
| Blinding | 11a | If done, who was blinded after assignment to interventions (for example, participants, care providers, those assessing outcomes) and how |  | Assess the risk of bias / ascertainment bias / performance bias / attrition bias / risk of unblinding (35); Prevent ambiguity / Who was blinded and how / reproducibility (8) | 3 | 35 |
|  | 11b | If relevant, description of the similarity of interventions |  | How similar are interventions? (24); Assures us that the blinding is effective / risk of bias is low / compromised blinding is reported (1) | 1 | 23 |
| Statistical methods | 12a | Statistical methods used to compare groups for primary and secondary outcomes |  | Assess the risk of reporting bias / only reporting "interesting" results) / assess the primary outcome and main comparisons remains the same (35); Assess the risk of multiplicity / bias of multiple tests / risk of false positive (5); Assess the appropriateness of the statistical methods, effect measure, significance level, and presentation (11) | 5 | 37 |
|  | 12b | Methods for additional analyses, such as subgroup analyses and adjusted analyses |  | Assess the risk of spurious findings in subgroup analysis / post hoc analyses or categorization are identified / assess risk of multiplicity (37); Assess the appropriateness for adjusted analysis and variables used / objective criteria to select variables for adjustment / ensures it is using baseline data rather than post-randomisation data (1) | 4 | 33 |
|  | 12b.1 |  | Was effect modification by age, sex, anthropometric status, and (if relevant to age of participants) gestation, birthweight, and breastfeeding status considered? | Interpretation / evaluation of the pediatric specific confounders that were accounted for (8); Methods used to address effect modification / were the methods chosen a priori (5) | 0 | 12 |
| Ethical considerations |  |  | Was information about research provided to children and assent taken (appropriate for age)? | Assess whether the process was appropriate for age / patient competence/ maturity/ understanding (24); Assess whether the process was ethical / patient voluntariness/ assent/ dissent/ autonomy (33); Role of parents or guardians/ proxy consent (7) | 3 | 38 |
|  |  |  | What measures were taken to reduce pain, distress, and invasiveness of research methods? | Readers are able to assess whether risk / harm was minimized / Nuremberg code (11); Assess whether pain / distress / invasiveness were minimized / parental access / pediatric specific measures (10) | 0 | 18 |
| Results | | | |  |  |  |
| Participant flow (a diagram is strongly recommended) | 13a | For each group, the numbers of participants who were randomly assigned, received intended treatment, and were analysed for the primary outcome |  | Transparency / clarity (37); Assess the risk of attrition bias / selection bias by not using all randomized participants (9); Prevents ambiguity of statistical methods through labels like "intention to treat" or "per protocol" (7); Generalizability / applicability / Number of participants assessed for eligibility? (6) | 2 | 36 |
|  | 13b | For each group, losses and exclusions after randomisation, together with reasons |  | Transparency / clarity / reporting of protocol violations or mistakes / allows synthesis of results (25); Assess the risk of attrition bias / selection bias by not using all randomized participants / assess the reasons for losses (16) | 2 | 33 |
| Recruitment | 14a | Dates defining the periods of recruitment and follow-up |  | Assess context / generalizability / applicability / reproducibility (35); Allows the reader to assess whether follow-up period was sufficient / pediatric population needs long follow-up (3) | 1 | 35 |
|  | 14b | Why the trial ended or was stopped |  | Assess the appropriateness of stopping guideline: processes and people involved / risk of stopping bias / prevent underreporting of early stopping (23); Assess the risk of research waste through futility or shown benefit without stopping / assess the risk of harms without stopping (1) | 1 | 22 |
| Baseline data | 15 | A table showing baseline demographic and clinical characteristics for each group |  | Generalizability / applicability / assess whether results are relevant to an individual patient / implementation (36); Assess whether there are possible confounders differing between groups / significance tests should not be performed (7) | 4 | 34 |
|  | 15.1 |  | Age distribution of children in the trial | Interpretation / allows evaluation of whether the findings of the trial are generalizable to their ages (24); Comparison of studies / systematic review (6) | 2 | 23 |
|  | 15.2 |  | Number of children in the trial by age categories (0-28 days, 1-12 months, 1-2 years, 3-5 years, 5-11 years, 12-17 years) | Allows evaluation of whether treatment was appropriate for age / age-related differences in treatment effect (22); Comparison of studies / systematic review (6); Allows evaluation of whether treatment was appropriate for subgroups / rationale for subgroups (6) | 3 | 21 |
|  | 15.3 |  | Distribution by sex, nutritional status, if relevant by gestation, birthweight, breastfeeding status, pubertal stages | Pediatric specific confounders (13); Age-baseline interactions / age related diagnostic issues (1) | 4 | 10 |
| Numbers analysed | 16 | For each group, number of participants (denominator) included in each analysis and whether the analysis was by original assigned groups |  | Assess the risk of attrition bias / selection bias by not using all randomized participants or non-random loss of participants (34); Prevents ambiguity of statistical methods through labels like "intention to treat" or "per protocol" (6); Assess the appropriateness of methods of handling missing data / Assess the robustness of the assumptions made for missing data (sensitivity analysis) (3) | 1 | 36 |
| Outcomes and estimation | 17a | For each primary and secondary outcome, results for each group, and the estimated effect size and its precision (such as 95% CI) |  | Allows assessment of selective reporting / reporting bias (36); Allows assessment of uncertainty of the results / p-values alone not sufficient / effect size important to report, because it might be significant, but not clinically important / relevance (9) | 5 | 33 |
|  | 17b | For binary outcomes, presentation of both absolute and relative effect sizes is recommended |  | Allows the reader to assess the effect / assess whether the size of the effect is relevant to clinical practice / relative risk allows generalizability (23); Allows the reader to assess the risk in context of the disease itself / effect size of near one might be important for common diseases, different for rare diseases (3) | 3 | 22 |
| Ancillary analyses | 18 | Results of any other analyses performed, including subgroup analyses and adjusted analyses, distinguishing pre-specified from exploratory |  | Allows assessment of selective reporting / reporting bias (37); Assess whether interactions were accounted for / interactions are reported with confidence intervals (2); Assess the risk of spurious findings in subgroup analysis / post hoc analyses or categorization are identified and justified / assess risk of multiplicity (6) | 2 | 35 |
| Harms | 19 | All important harms or unintended effects in each group (for specific guidance see CONSORT for harms) |  | Enables balanced assessments of the results / assess acceptability and usefulness (34); Absolute risk of each adverse event must be reported / definitions should be reported, so their appropriateness can be assessed (3); Prevents common bias of under-reporting harms / ethical responsibility (7) | 4 | 33 |
|  | 19.1 |  | Results of plan for long-term adverse reactions, particularly those related to growth and development. If not, rationale for why not | Effects on development (38); Pediatric-specific issues in follow-up / long-term follow-up needed (12); Rare events / sustainability (8) | 2 | 47 |
| Discussion |  |  |  |  |  |  |
| Limitations | 20 | Trial limitations, addressing sources of potential bias, imprecision, and, if relevant, multiplicity of analyses |  | Enables balanced assessments of the results / assess acceptability and usefulness / transparency (35); Assess whether appropriate measures were used to contain bias, imprecision, or prevent spurious findings (3) | 2 | 35 |
| Generalisability | 21 | Generalisability (external validity, applicability) of the trial findings |  | Allows reader to assess whether the results apply to their participants / implementation / applicability (36); Enables balanced assessments of the results / assess acceptability and usefulness / transparency (4) | 1 | 37 |
| Interpretation | 22 | Interpretation consistent with results, balancing benefits and harms, and considering other relevant evidence |  | Assess the results in context / relevance / consistency with previous results (36); Assess the confidence of the results / Bayesian methods to aid interpretation (2) | 2 | 34 |
| Other Information | | |  |  |  |  |
| Registration | 23 | Registration number and name of trial registry |  | Scientific, ethical and moral responsibility to register / assess risk of publication bias / selective reporting bias / underreporting (25); Basic information available to the public / allows studies to be found (5); Clarity / transparency / ability to distinguish multiple reports of the same trial / avoids duplication of research and publication / research waste (5) | 8 | 19 |
| Protocol | 24 | Where the full trial protocol can be accessed, if available |  | Allows assessment of what aspects of the methods were a priori, and what were post hoc / allows assessment of changes to the protocol (24); Allows assessment of presence of selective reporting of outcomes (4) | 1 | 23 |
| Funding | 25 | Sources of funding and other support (such as supply of drugs), role of funders |  | Assess potential competing interests / transparency / assess whether reimbursement amount is reasonable / risk of bias (reporting / publication bias) (26) | 4 | 22 |
