# Supplementary material for: Recommendations and evidence for reporting items in pediatric clinical trial protocols and reports: two systematic reviews
Source: Trials. 2015 Sep 18;16:417. doi: 10.1186/s13063-015-0954-0 (PMC4574457; doi:10.1186/s13063-015-0954-0)
Supplement: Additional file 7: — Qualitative analysis: evidence from the literature organized by meaning and content, presented by descriptive themes, and sorted into analytic themes. (PDF 191 kb) [file 13063_2015_954_MOESM7_ESM.pdf]

Qualitative analysis: evidence from the literature organized by meaning and content, presented by descriptive themes and sorted into analytic themes.

External validity

Themes of evidence were considered part of the over-arching theme of external validity if the author described that the reporting of this item allows readers to assess:
•relevance of the trial to their needs
•context of the trial
•the applicability of the trial
•generalizability of the trial to their population
•scope of the eligibility criteria is reasonable (not too narrow)
•interventions are generalizable to the real world
•whether the interventions are apply to other settings and participants / patients / ages
•whether the results apply to other settings and participants / patients / ages

Reporting bias

Themes of evidence were considered part of the over-arching theme of reporting bias if the author described that the reporting of this item:
•discourages reporting bias
•discourages selective reporting bias
•allows readers to assess whether all results / harms / outcomes were reported
•allows readers to distinguish post hoc revisions
•allows readers to identify discrepancies between protocol and report
•allows readers to assess whether pre-specified primary outcome and main comparisons are mirrored in the report
•allows readers to assess whether clinically important effect sizes or equivalence margins were specified a priori

Internal validity

Themes of evidence were considered part of the over-arching theme of internal validity if the author described that the reporting of this item allows readers to assess:
•potential for bias through competing interests or influence
•the appropriateness of sample size calculation, trial design, and statistical analyses
•the risk of co-intervention bias
•the risk of confounding bias
•the risk of ascertainment bias
•the risk of selection bias
•the rationale for each assumption or component in analysis
•whether clustering is accounted for
•the risk of predictability through non-random procedures
•the risk of biased effect size
•the risk of allocation concealment corruption
•the risk of attrition bias
•the risk of unblinding
•the validity of data collection methods in selected age groups
•the validity of instruments in selected age groups
•the appropriateness of variables in adjusted analysis
•the appropriateness of methods for handling missing data
•the appropriateness of interim analyses
•whether age-related differences in treatment effect, baseline, or diagnostics are accounted for
•whether effect modification was accounted for a priori
•whether interactions were accounted for
•whether appropriate measures were used to contain bias, imprecision, or prevent spurious findings

Accountability

Themes of evidence were considered part of the over-arching theme of accountability if the author described that the reporting of this item:
•allows readers to assess who is accountable for which aspects of the trial
•allows recognition for all contributions to the trial
•reduces ghost authorship
•allows readers to assess the appropriateness of those selected for their roles
•allows readers to assess the expertise of those selected
•allows readers to assess the mandate of those selected
•allows readers to assess whether a statistician was included
•clarifies the roles and responsibilities of all involved
•allows readers to assess whether researchers are fulfilling their responsibilities to participants / parents / children
•allows readers to assess the appropriateness of the people involved in interim analyses and stopping guidelines
•allows readers to assess whether researchers are reachable

Transparency

Themes of evidence were considered part of the over-arching theme of transparency if the author described that the reporting of this item allows readers to assess:
•oversight and review
•potential competing interests
•transparency
•the motivation for the trial
•the independence of the data monitoring committee
•the independence of researchers from sponsors
the independence of the adverse event monitoring, reporting and management procedures
•whether all relevant parties are independent
•the appropriateness of the role of the sponsors
•possible conflicts of interest
•the independence of the quality and safety assurance procedures

Usefulness

Themes of evidence were considered part of the over-arching theme of usefulness if the author described that the reporting of this item:
•ensures the publication is searchable and findable
•makes the information consistent with other publications
•ensures the information is useful
•structures the publication consistently with other publications
•reduces research waste
•prevents duplication of research
•allows gaps in knowledge to be identified
•allows researchers to synthesize the information in meta-analyses and systematic reviews
•allows reader to assess whether a core outcome set was used
•ensures the research is being built upon previous research
•ensures the research is consistent with previous research
•allows readers to compare the results to those of other studies
•allows readers to assess the risk of limited access preventing the trial from independent validation
•allows readers to assess whether the research will be wasted through futility
•allows readers to assess the ability of others to verify the validity of the trial results
•makes the age subgroups consistent with other publications

Reproducibility

Themes of evidence were considered part of the over-arching theme of reproducibility if the author described that the reporting of this item allows readers to:
•understand exactly what was done for “standard care” or “usual care”
•assess whether it would be feasible to reproduce the trial or intervention
•objectively reproduce all aspects of the intervention
•fully reproduce all aspects of the trial and analysis
•assess the risk of ambiguity
•understand what is meant by per-protocol or intention-to-treat

Interpretability

Themes of evidence were considered part of the over-arching theme of interpretability if the author described that the reporting of this item:
•enables reader to make balanced assessments of the results
•aids the proper interpretation of the results
•enables reader to interpret methods
•ensures the publication is understandable
•allows readers to assess the risk in context of the disease itself
•allows readers to assess the confidence of the results
•allows readers to understand the limits upon the results
•enables readers to evaluate which procedure is safest
•enables readers to evaluate which procedure is best
•allows readers to properly interpret the age specific context of the results
•enables readers to evaluate the clinical importance of the results
•allows readers to interpret whether the groups truly differ

Research ethics

Themes of evidence were considered part of the over-arching theme of ethics if the author described that the reporting of this item:
•allows funders and ethics boards to assess the ethical justification
•discourages unnecessary research
•ensures the inclusion of children is justified
•allows the assessment of the ethical appropriateness of the comparators
•allows the ethical oversight to be assessed
•allows external review of participant burden
•ensures the minimal number of participants included to meet objectives
•allows readers to assess the appropriateness of the procedures to protect the safety of the participants
•allows readers to assess adherence to regulations and policies
•ensures the protection of children’s health information
•allows readers to assess whether equipoise will be met throughout the trial & stopped when violated
•ensures that ethical approval is sought
•allows readers to assess whether informed consent and assent procedures were appropriate
•allows readers to assess whether proxy consent was appropriate
•allows readers to assess whether the changing consent ability
•allows readers to assess whether the process to protect confidentiality of personal information from outsiders is appropriate
•allows readers to assess whether confidentiality of children from parents is appropriately considered
•allows readers to assess whether compensation plans are appropriate
•allows readers to assess whether interventions are available to participants following the study
•allows readers to assess whether the extrapolation from existing data was considered and used
•allows readers to assess whether the question could be answered using existing data
•allows readers to assess whether risks were minimized
•allows readers to assess whether follow-up was sufficient to capture developmental and long-term harms
•allows readers to assess whether patient autonomy was assured
•ensures there is sufficient evidence for the efficacy of the intervention in the selected population to justify the risk

Publication bias

Themes of evidence were considered part of the over-arching theme of publication bias if the author described that the reporting of this item:
•aids literature searches
•makes the document more easily found or linked
•allows readers to assess whether the study is likely to have publication restrictions or delays
•allows readers to make health care decisions if they do not have access to full-text
•allows readers to assess the risk of publication bias
•allows readers to assess whether the publication will be available to the public

Scientific soundness

Themes of evidence were considered part of the overall theme of scientific soundness if the author described that the reporting of this item allows the reader to assess:
•the expertise of those responsible
•the scientific appropriateness of the comparators
•the purpose of the trial
•the scientific soundness of sample size calculation, trial design, and statistical analyses
•the sample size calculations for the primary outcome
•possible multiplicity
•factors used for stratification, blocking, or minimisation for scientific relevance
•adequacy of the allocation mechanism
•scientific soundness of data collection methods
•scientific soundness of measurement instruments
•processes to enhance data quality
•the risk of false positives
•the appropriateness of the statistical methods, effect measure, significance level, and presentation
•the scientific soundness of criteria to select variables for adjustment
•the robustness of the assumptions made for missing data (sensitivity analysis)
•the scientific soundness of the scientific justification for the trial
•the scientific soundness of extrapolation from existing data
•the justification of the age group used
•the rationale for sub-groups
•what is known about pediatric pharmacokinetics
•the scientific soundness of intervention dose/duration/strength/route of administration
•whether the rationale matches the methods
•the scientific soundness of the selected margin for non-inferiority or equivalence
•the scientific justification for the clinically relevant effect size
•whether the evidence for efficacy is conclusive
